# Hair analysis for New Psychoactive Substances (NPS): Still far from becoming the tool to study NPS spread in the community?

**DOI:** 10.1016/j.toxrep.2021.09.003

**Published:** 2021-09-28

**Authors:** Dimitra Florou, Vassiliki A. Boumba

**Affiliations:** Department of Forensic Medicine and Toxicology, Faculty of Medicine School of Health Sciences, University of Ioannina, University Campus, 45110 Ioannina, Greece

**Keywords:** New Psychoactive Substances, Hair analysis, Extraction, Mass spectrometry, LOD, Identification

## Abstract

In this review article, we performed an overview of extraction and chromatographic analysis methods of NPS in hair from 2007 to 2021, evaluating the limit of detection (LOD), limit of quantification (LOQ), limit of reporting (LOR), and limit of identification (LOI) values reported for each NPS. Our review aimed to highlight the limitations of modern hair analytical techniques, and the prerequisites for the proper evaluation and use of analytical results in relation to the objectives of NPS hair analysis. In the selected studies the detection of a total of 280 NPS was reported. The detected NPS belonged to seven classes: synthetic cannabinoids with 109 different substances, synthetic opioids with 58, cathinones with 50, phenethylamines with 34, other NPS with 15, tryptamines with ten, and piperazines with four substances. The NPS hair analysis of real forensic/ clinical cases reported the detection of only 80 NPS (out of the 280 targeted), in significantly higher levels than the respective LODs. The analytical protocols reviewed herein for NPS hair analysis showed continuously growing trends to identify as many NPS as possible; the extraction methods seem to have a limited potential to improve, while the various mass spectroscopic techniques and relevant instrumentation provide an enormous field for development and application. Hair is a biological indicator of the past chronic, sub-chronic, and, even, in certain cases, acute exposure to xenobiotics. Therefore, future research in the field could progress NPS hair analysis and aim the monitoring of NPS expansion and extent of use in the community.

## Highlights

• The detection of 280 NPS has been reported to be enabled through hair analysis.
• The LODs/ LOQs for these NPS are as low as pg/mg of hair.
• The NPS hair concentrations in clinical/forensic samples are considerably higher than the respective LOD.
• Untargeted-mass spectroscopic detection techniques could advance NPS hair analysis.
• NPS hair analysis could become the tool to monitor the extent of NPS use worldwide.

## Graphical abstract

**Figure:**
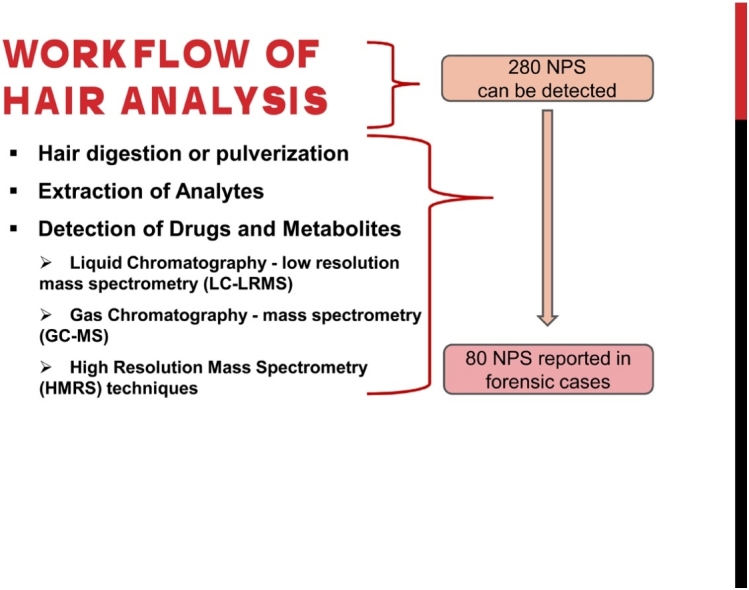

## 1 Introduction

Over the last few years, new recreational psychotropic substances, have been synthesized and flooded the illicit drug market, being identified under different labels e.g. “legal highs,” “research chemicals,” or “designer drugs”. Nowadays, they are known as “Novel Psychoactive Substances” (NPS). Initially, NPS were designed to mimic the effects of internationally controlled illegal drugs while being structurally different to not be controlled under the Misuse of Drugs Act 1971 [1]. Currently, there are more than 1000 NPS belonging to defined groups, e.g. synthetic cannabinoids, phenethylamines, cathinones, piperazines, plant-based substances, and miscellaneous substances including hallucinogens, synthetic opioids, and synthetic benzodiazepines [1].

NPS have become a worldwide health problem due to the vast variety of novel substances available, their ambiguous legal situation and ability to pass undetected routine toxicological, immunochemical tests, their rapid adaptation to legal restrictions, and their unidentified, in many cases, adverse effects [2,3]. Most of these products are more pharmacologically potent and hazardous than classical drugs of abuse [4]. Meanwhile, several fatal and acute intoxication cases have been accredited to this diverse group of compounds [[5], [6], [7]].

Hair analysis can be used in biomonitoring of toxicants and it is the method of choice for assessing retrospective evaluation of the past, chronic, sub-chronic, and, even, in certain cases, acute exposure to xenobiotics [8]. The past detection window can exceed up to several months or even years, while segmental hair analysis has been used to provide information on the history and state of drug abuse of the tested individual [8,9]. While generally, the biomonitoring of particular toxicants applied in clinical studies [10,11], hair analysis was performed, specifically, to study the exposure of certain populations to pesticides and other organic pollutants [[12], [13], [14], [15]] and, the prevalence of NPS among drug users or addicts [9,[16], [17], [18]]. We are of opinion that when large populations would be subjected to NPS hair analysis, then the relevant gathered results would provide epidemiological data on the NPS trends and extent of use in the community.

In this review article, we performed an overview of extraction and chromatographic analysis methods of NPS in hair from 2007 to 2021, evaluating the limit of detection (LOD), limit of quantification (LOQ), limit of reporting (LOR), and limit of identification (LOI) values of each NPS. Our review aimed to highlight the limitations of modern hair analytical techniques, and the prerequisites for the proper evaluation and use of analytical results in relation to the objectives of the analysis.

The following keywords: “New Psychoactive Substances” or “NPS” and “hair” and “analytical methods” and “pre-treatment methods” or “extraction methods” and “phenethylamines” or “piperazines” or “synthetic cannabinoids” or “cathinones” or “synthetic opioids”, or “tryptamines”, were used to search the databases Scopus and PubMed. Information of interest of this review were found in 45 reports which were selected for further study.

## 2 Extraction and detection methods for the determination of NPS in hair

In the selected studies the detection of a total of 280 NPS was reported, for the different hair analysis protocols. The detected NPS belonged to seven classes: phenethylamines (2C-X series, other phenethylamines), piperazines, synthetic cathinones (SCa), synthetic cannabinoids (SCs, categorized in the subclasses of benzoylindoles, naphthoylindoles, phenylacetylindoles, naphthoypyrroles, other SCs), synthetic opioids (SO), tryptamines, and other NPS classes. Synthetic cannabinoids dominated the other categories with 109 different substances, followed by synthetic opioids with 58, cathinones with 50, phenethylamines with 34, other NPS with 15, tryptamines with ten, and piperazines with four substances. The overview of methods of extraction, analysis and detection to determine NPS in hair are exhibited in Table 1, Table 2, Table 3, Table 4, Table 5.

**Table 1:** Selected parameters of hair analysis for Synthetic Phenethylamines and Piperazines.

**NPS**	**Extraction Method**	**Method of Analysis**	**LOD (pg/mg)**	**LOQ** **(pg/mg)**	**LOI/** **LOR** **(pg/mg)**	**Concentrations- Clinical/forensic samples (pg/mg)**	**References**
**PHENETHYLAMINES (2C-x-series)**							
2 C-B	MeOH/ HCL 0.25 M at 50 °C	GC/MS	4	20			[19]
	MeOH at 55 °C	LC-MS/MS	6.2	12			[20]
	MeOH/ HCL 0.1 M at 40 °C, pulverization	LC-MS/MS	50		-/100		[21]
	Enzymatic Digestion, LLE neutral and basic with DEE/EA	LC-MS/MS	80				[22]
2 C-D	Enzymatic Digestion, LLE neutral and basic with DEE/EA	LC-MS/MS	50				[22]
2 C–E	MeOH/ HCL 0.1 M at 40 °C, pulverization	LC-MS/MS	10		-/100		[21]
	Enzymatic Digestion, LLE neutral and basic with DEE/EA	LC-MS/MS	20				[22]
2 C–I	MeOH/ HCL 0.1 M at 40 °C, pulverization	LC-MS/MS	10		-/100		[21]
	Enzymatic Digestion, LLE neutral and basic with DEE/EA	LC-MS/MS	80				[22]
2 C-G	MeOH/ HCL 0.1 M at 40 °C, pulverization	LC-MS/MS	10		-/100		[21]
2 C-N	MeOH/ HCL 0.1 M at 40 °C, pulverization	LC-MS/MS	50		-/100		[21]
2 C–P	MeOH at 55 °C	LC-MS/MS	1	2			[20]
	Enzymatic Digestion, LLE neutral and basic with DEE/EA	LC-MS/MS	50				[22]
2 C–T	Enzymatic Digestion, LLE neutral and basic with DEE/EA	LC-MS/MS	100				[22]
2C–T-4	Acid digestion at 45 °C, PLE, SPE/C18 cartridge	LC-HRMS	10	50			[23]
Me-EPHE	MeOH/ HCL 0.1 M at 40 °C, pulverization	LC-MS/MS	10		-/100		[21]
MXP	MeOH/ HCL 0.1 M at 40 °C, pulverization	LC-MS/MS	1		-/100		[21]
N-EPHE	MeOH/ HCL 0.1 M at 40 °C, pulverization	LC-MS/MS	50		-/100		[21]
PE	MeOH/ HCL 0.1 M at 40 °C, pulverization	LC-MS/MS	50		-/100		[21]
PS-EPHE	MeOH/ HCL 0.1 M at 40 °C, pulverization	LC-MS/MS	10		-/100		[21]
25B-NBOMe	MeOH at 55 °C	LC-MS/MS	4.1	8.2			[20]
25C-NBOMe	MeOH/ HCL 0.1 M at 40 °C, pulverization	LC-MS/MS	1		-/100		[21]
	MeOH at 55 °C	LC-MS/MS	1.5	3			[20]
25H-NBOMe	MeOH at 55 °C	LC-MS/MS	1	2			[20]
25I-NBOMe	MeOH/ HCL 0.1 M at 40 °C, pulverization	LC-MS/MS	1		-/100		[21]
	MeOH at 55 °C	LC-MS/MS	1.5	3			[20]
**OTHER PHENETHYLAMINES- AMPHETAMINE TYPE**							
Butylone (bk-MBDB)	SPE/Bond Elute Certify I	LC-MS/MS	0.8	1			[24]
	MeOH at 55 °C	LC-MS/MS	3.7	7.4			[20]
	0.1 M HCOOH at 45 °C	LC-MS/MS	5	20			[25]
	MeOH/ACN/aq. HCOONH_4_ at 40 °C	LC-MS/MS	8	25			[26]
	MeOH, MeOH/HCL 33 %	LC-MS/MS	10				[27]
	MeOH/ HCL 0.1 M at 40 °C, pulverization	LC-MS/MS	10		-/100		[21]
DMA	MeOH/ HCL 0.1 M at 40 °C, pulverization	LC-MS/MS	10		-/100		[21]
MDEA	MeOH/ HCL 0.1 M at 40 °C, pulverization	LC-MS/MS	1		-/100		[21]
	PLE, SPE/C18 cartridge	LC-MS-MS	1	4.5			[28]
	MeOH/ HCL 0.25 M at 50 °C	GC/MS	24	80		100−25,000	[19]
MPHP	MeOH/ HCL 0.1 M at 40 °C, pulverization	LC-MS/MS	1		-/100		[21]
	M3® reagent at 100 °C	LC-MS/MS	5	20			[29]
MXE	MeOH at 55 °C	LC-MS/MS	1	2		7.7−27	[20]
	Acid digestion at 45 °C, PLE, SPE/C18 cartridge	LC-HRMS	3	10			[23]
	Incubation at 95 °C, LLE with Hept/EA, DCM/Isopropanol	LC-HRMS- Orbitrap	5		5/-		[30]
	MeOH/ HCL 0.1 M at 40 °C, pulverization	LC-MS/MS	10		-/100		[21]
PMA	0.1 M HCOOH at 45 °C	LC-MS/MS	5	10			[25]
	MeOH at 55 °C	LC-MS/MS	8.8	18			[20]
	Incubation at 45 °C, SPE/MCX® Oasis cartridge	LC-MS/MS	10	50			[31]
	MeOH/ HCL 0.1 M at 40 °C, pulverization	LC-MS/MS	10		-/100		[21]
	MeOH/ HCL 1%	GC/MS	250	500		20,100	[32]
PMMA	MeOH at 55 °C	LC-MS/MS	1.3	2.6			[20]
	MeOH/ HCL 0.1 M at 40 °C, pulverization	LC-MS/MS	10		-/100		[21]
PPMA	MeOH/ HCL 0.1 M at 40 °C, pulverization	LC-MS/MS	10		-/100		[21]
4-FA	MeOH at 55 °C	LC-MS/MS	1.6	3.2			[20]
	0.1 M HCOOH at 45 °C	LC-MS/MS	2	5			[25]
	MeOH, MeOH/HCL 33 %	LC-MS/MS	10				[27]
4-FMA	MSPE: MeOH/ HCL 0.1 M at 60 °C, SPE/ C18	LC-MS/MS	2	10			[33]
4-MTA	0.1 M HCOOH at 45 °C	LC-MS/MS	2	20			[25]
	Incubation at 45 °C, SPE/MCX® Oasis cartridge	LC-MS/MS	20	50			[31]
5-APB	M3® reagent at 100 °C	LC-MS/MS	5	20			[29]
	MeOH/ HCL 0.1 M at 40 °C, pulverization	LC-MS/MS	10		-/100		[21]
5-EAPB	M3® reagent at 100 °C	LC-MS/MS	5	20			[29]
5-MAPB	MeOH at 55 °C	LC-MS/MS	4.6	9.2			[20]
6-APB	M3® reagent at 100 °C	LC-MS/MS	5	20		70	[29]
	MeOH/ HCL 0.1 M at 40 °C, pulverization	LC-MS/MS	10		-/100		[21]
	MeOH at 55 °C	LC-MS/MS	17	35			[20]
6-MAPB	M3® reagent at 100 °C	LC-MS/MS	5	20			[29]
**PIPERAZINES**							
Benzylpiperazine	0.1 M HCOOH at 45 °C	LC-MS/MS	5	20			[25]
	MeOH, MeOH/HCL 33 %	LC-MS/MS	10				[27]
	MeOH/ HCL 0.1 M at 40 °C, pulverization	LC-MS/MS	50		-/100		[21]
mCPP	MeOH at 55 °C	LC-MS/MS	3	6			[20]
	0.1 M HCOOH at 45 °C	LC-MS/MS	5	20			[25]
	MSPE: MeOH/ HCL 0.1 M at 60 °C, SPE/ C18	LC-MS/MS	5	10		3,411.4- >4000	[33]
	Incubation at 95 °C, LLE with Hept/EA, DCM/Isopropanol	LC-HRMS- Orbitrap	5		500/-		[30]
	MeOH, MeOH/HCL 33 %	LC-MS/MS	10				[27]
	Incubation at 45 °C, SPE/MCX® Oasis cartridge	LC-MS/MS	10	50			[31]
	MeOH/ACN/aq. HCOONH_4_ at 40 °C	LC-MS/MS	25	37			[26]
	MeOH/ HCL 0.1 M at 40 °C, pulverization	LC-MS/MS	50		-/100		[21]
	MSPE: Basic digestion at 50 °C, SPE/MCX® cartridges	GC-MS	–	LLOQ: 50			[34]
MeOPP	MeOH/ACN/aq. HCOONH_4_ at 40 °C	LC-MS/MS	29	46			[26]
	MeOH/ HCL 0.1 M at 40 °C, pulverization	LC-MS/MS	50		-/100		[21]
	MSPE: Basic digestion at 50 °C, SPE/MCX® cartridges	GC-MS	–	LLOQ:50			[34]
TFMPP	MSPE: MeOH/ HCL 0.1 M at 60 °C, SPE/ C18	LC-MS/MS	1				[33]
	MeOH/ACN/aq. HCOONH_4_ at 40 °C	LC-MS/MS	9	24			[26]
	MeOH, MeOH/HCL 33 %	LC-MS/MS	10				[27]
	MeOH/ HCL 0.1 M at 40 °C, pulverization	LC-MS/MS	50		-/100		[21]
	MSPE: Basic digestion at 50 °C, SPE/MCX® cartridges	GC-MS	–	LLOQ: 50			[34]

Generally, the analytical methodology consisted of the following steps: hair decontamination from external contaminants, hair digestion or pulverization and analytes extraction from a hair amount ranging between 10−100 mg. The hair decontamination procedures used were washing with: (i) organic solvents, such as methanol [20,21,23,24,32,34,43,44,47,50,51,53,54,56,58,60], ethanol [35], acetone [19,22,[25], [26], [27],^37^,^38^,^41^,^46^,^49^,^57^,^61^,^63^], hexane [27], petroleum ether [41,49], dichloromethane [20,21,23,24,[28], [29], [30], [31],^33^,^34^,^36^,^39^,^40^,^42^,^45^,^48^,[52], [53], [54], [55], [56],[58], [59], [60],^62^], isopropanol [23,28], isooctane [38,52]; (ii) sodium dodecyl sulfate solution [35,46]; (iii) non-ionic surfactant and emulsifier (TWEEN 80) [25,37,63]; and (iv) water of variable analytical grade: distilled [19,25,32,35,37,43,44,46,47,50,51,63], deionized [31,34,41,49], ultra-pure [36,45,55,57] and not specified type of water [22,23,26,26,27,30,59,62].

Analytes’ extraction from hair, which followed hair digestion or pulverization, was achieved by either single step methanol extraction [20,25,27,43,44,47,[49], [50], [51],^53^,^56^,[58], [59], [60],^62^], or acidified methanol extraction [19,21,32,49], or ethanol extraction [41], or liquid-liquid extraction (LLE) with various mixtures of organic solvents [22,25,30,36,39,40,42,45,46,48,55,61,63], or extraction with aqueous buffers of organic solvents (methanol/ acetonitrile/ ammonium formate) [26], or (methanol/ acetonitrile/ trifluoroacetic acid (TFA)) [37] or (methanol/ TFA) [38] or (methanol/ acetonitrile/ ammonium acetate) [57], or solid phase extraction (SPE) on various cartridges [24,29,31,52,54] or mixed-mode solid phase extraction (MSPE) [33,34] or pressurized liquid extraction (PLE) [23,28,35], assisted in many cases by mild heat of the samples [[19], [20], [21], [22], [23],^25^,^26^,^29^,^34^,^36^,^39^,^40^,[42], [43], [44], [45],^47^,^48^,[50], [51], [52], [53], [54], [55], [56],[58], [59], [60],^62^,^63^]. Most of the reports presented the concurrent detection of several NPS from different chemical classes [[19], [20], [21], [22], [23],[25], [26], [27], [28], [29], [30], [31],^33^,^46^,^62^] and others focused on the analysis of just one NPS class [24,32,34,[43], [44], [45],[47], [48], [49], [50], [51], [52], [53], [54], [55], [56], [57], [58], [59], [60], [61],^63^]. Most of the reviewed methodologies used liquid chromatography coupled to low resolution mass spectrometry [21,22,[24], [25], [26], [27], [28], [29],^31^,^33^,^35^,^36^,^39^,^41^,[43], [44], [45],[47], [48], [49], [50], [51], [52], [53], [54], [55], [56], [57],[60], [61], [62]] followed by gas chromatography-mass spectrometry [19,32,34,40,46], and more recently by techniques coupled to high resolution mass spectrometry techniques (HMRS) [23,30,37,38,58,59,62,63] for the detection of drugs and metabolites. The selection and application of the appropriate NPS extraction method from hair was intimately bound to the properties of the chemical examined, the sensitivity of the detection instrument, and the hair amount. It is generally accepted that a rapid and efficient extraction is essential for forensic laboratories and the justice timeline, allowing the minimization of false-negative results and the maximum sensitivity of detection (lower LODs/LOQs).

From analytical point of view, the LOD and LOQ are defined with strict and widely accepted criteria [64]. All but one of the reviewed manuscripts reported LODs and LOQs at the level of nanogram or picogram NPS per milligram of hair, while the exception attained NPS levels at nanogram per 10-mm hair segment [35]. In addition, the limit of reporting (LOR) was another relevant parameter defined as the concentration for reporting positive samples, aiming to discriminate the active drug incorporation during consumption from the deposition of NPS on hair during external exposure [21]. LOR values have been set (at the level of 100 pg/mg of hair) being at least 10fold higher than the respective LODs for most of the 132 NPS analysed. It is obvious that such a value can only be set arbitrarily. Moreover, the limit of identification (LOI) has been also utilized in one study and defined as the lowest analyte concentration that could be correctly identified by the screening software [30] and it was equal to up to a hundred times higher than the respective LODS for the 10 NPS applied. The efforts to set LOR or LOI values to report NPS in hair are indicative of the concerns about the subsequent proper interpretation of the hair analysis results and to discriminate positive hair samples due to NPS active use from passive exposure.

### 2.1 Determination of synthetic phenethylamines and piperazines in hair

Synthetic phenethylamines that share a common phenethylamine moiety are considered to be a noteworthy group of legal highs [1]. Psychedelic phenethylamines such as 2C (2C-*x*) have methoxy groups on the two and five positions of a benzene ring, and various lipophilic substituents at position four. NBOMe (or 25X-NBOMe) is another class containing an *N*-(2-methoxy) benzyl substituent. Additionally, other phenethylamines, such as PMMA, include designer drugs from the amphetamine class, which hold serotonergic effects.

**2C-x series:** A total of five studies have been carried out for the determination of eighteen 2 C-x in hair. LC–MS/MS [[20], [21], [22]] assays have mainly been utilized, while GC/MC [32] and LC-HRMS [23] have been used to determine only 2 substances. The relative data are presented in Table 1.

Three of these studies [[21], [22], [23]] have been engaged with the simultaneous analysis of phenethylamines with other NPS classes. The dominant extraction method applied is acidified methanol with various HCL concentrations (0.1 M [21] or 0.25 M [19]), to define 12 2C-x. LLE with a diethyl ether-ethyl acetate mixture was used to define 6 analytes, before their LC–MS/MS analysis [22] resulting to a higher LOD for 2 C-B, 2 C–E, and 2 C–I, compared to their extraction with acidified methanol [21].

The LODs achieved for 25C-NBOMe and 25I-NBOMe were comparable, after methanol [20] or acidified methanol extraction [21]. Markedly, 2 C–P provided an admittedly low LOD after methanol extraction [20], comparing to that obtained after other LLEs [22].

The most elaborated extraction method used was a PLE followed by SPE to determine 2 C–T-4 by LC-HRMS/MS analysis. The respective LOD attained was comparable to that achieved for other NPS of this class detected with LC-HRMS [23].

Unexpectedly, the LODs achieved with GC–MS methods after extraction with acidified methanol were lower in most cases than the respective with LC–MS methods.

**Other Synthetic Phenethylamines:** A total of fourteen studies have been interpreted and the relative data are presented in Table 1, including 16 different amphetamine type-phenethylamines in hair. Detection methods included: LC–MS/MS [20,21,[24], [25], [26], [27], [28], [29],^31^,^33^], GC/MS [19,32], and LC-HRMS [23,30].

Ten of these studies [21,23,[25], [26], [27], [28], [29], [30], [31],^33^], report on their simultaneous analysis with NPS from other classes.

The principal extraction method applied was acidified methanol with various HCL concentrations (either 0.1 M [21] or 0.25 M [19] or 1% [32]), to define 10 substances. Single-step methanol extraction was used before the LC–MS/MS analysis of seven compounds (PMA, PMMA, MXE, 4-FA, 6-APB, butylone, and 5-MAPB) resulting in LODs comparable to those achieved with acidified methanol. However, acidified methanol [27] was less effective than the extraction with methanol [20], for the extraction of 4-FA and butylone.

Obviously, the concentration of HCl in methanol for extraction had a variable effect on the respective LODs of different NPS analysed with LC–MS/MS or GC/MS (e.g. extraction of PMA and MDEA with 1% or 0.25 M HCl in methanol [19,32] before GC/MS analysis, resulted in an excessively high LOD, as compared to that achieved with 0.1 M HCl in methanol and LC–MS/MS analysis [21]).

Various SPE protocols were applied to extract PMA, 4MTA, MXE, MDEA, 4FMA from hair [23,24,28,30,31,33] resulting in LODs comparable to other simpler protocols and detection with LC–MS or HRMS. Only the extraction of butylone with SPE resulted in considerably lower LOD compared to other applied extractions.

**Synthetic Piperazines:** Piperazines belong to a broad class of chemical compounds that have been designed to replicate the effects of ecstasy. Piperazines may act as central nervous system stimulants and can produce hallucinogenic or toxic effects similar to amphetamine and other sympathomimetics [1].

A total of nine studies report the determination of four piperazines in hair, using LC–MS/MS [20,21,[25], [26], [27],^31^,^33^], GC–MS [34], and LC-HRMS [30]. The relative data are presented in Table 1. Only two of these studies [20,34], report analysis of piperazines alone. Overall, the highest LODs for the four piperazines were achieved after extraction with acidified methanol and LC–MS/MS analysis [21]. Other LLE protocols, with methanol as the main solvent, resulted in comparable LODs. GC–MS methods were less sensitive, in terms of LLOQs, than LC–MS methods for mCPP, TFMPP, and MeOPP [34].

### 2.2 Determination of synthetic cathinones (SCa) in hair

Synthetic cathinones are stimulants, which belong to a category of drugs frequently recognised as bath salts [1,65]. These synthetic substances are chemical analogs of cathinone, the active stimulant of the khat plant, which act as monoamine release or reuptake inhibitors and have similar effects to amphetamines. In general, the polarity of these substances is increased by the β-keto group if compared to related amphetamines.

A total of 17 studies have been carried out for the determination of 50 SCa in human hair by LC–MS/MS [20,21,[24], [25], [26], [27],^29^,^33^,^35^,^36^,^39^], GC–MS [19,40], and LC-HRMS [23,30,37,38]. The relative data are presented in Table 2. Eleven of these studies [19,21,23,[25], [26], [27],^29^,^30^,^33^,^38^], have been engaged with the simultaneously analysis of cathinones with other NPS classes.

**Table 2:** Selected parameters of hair analysis for Synthetic Cathinones.

**NPS**	**Extraction Method**	**Analysis Method**	**LOD**	**LOQ**	**LOI/ LOR**	**Concentrations- Clinical/forensic samples (pg/mg)**	**References**
**CATHINONES**							
			**pg/mg**				
Amfepramone	MeOH at 55 °C	LC-MS/MS	4	8			[20]
	MeOH/ACN/aq. HCOONH_4_ at 40 °C	LC-MS/MS	13	40			[26]
	MeOH/ HCL 0.1 M at 40 °C, pulverization	LC-MS/MS	50		-/100		[21]
a-PBP	Basic digestion, SPE/Extrelut column	LC-MS	0.02 ng/ 10-mm	0.05 ng/10- mm			[35]
	MeOH/ACN/aq. HCOONH_4_ at 40 °C	LC-MS/MS	6	17			[26]
	MeOH/ HCL 0.1 M at 40 °C, pulverization	LC-MS/MS	10		-/100		[21]
α-PHP	SPE/Bond Elute Certify I	LC-MS/MS	0.1	1		4700.0/ 0−2.5 cm3600.0/ 2.5 −5 cm	[24]
a- PPP	MeOH/ HCL 0.1 M at 40 °C, pulverization	LC-MS/MS	10		-/100		[21]
	MeOH/ACN/aq. HCOONH_4_ at 40 °C	LC-MS/MS	13	25		α-PPP < LOQ	[26]
a-PVP	Basic digestion, SPE/Extrelut column	LC-MS	0.02 ng/ 10mm	0.05 ng/ 10-mm		0.8−1.2 ng/10- mm	[35]
	SPE/Bond Elute Certify I	LC-MS/MS	0.3	1		52.8/ 0−2.5 cm 24.4/ 2.5 −5 cm	[24]
	MeOH at 55 °C	LC-MS/MS	2	4			[20]
	Acid digestion at 45 °C, PLE, SPE/C18 cartridge	LC-HRMS	5	10			[23]
	MeOH/ HCL 0.1 M at 40 °C, pulverization	LC-MS/MS	10		-/100		[21]
	MeOH/ACN/aq. HCOONH_4_ at 40 °C	LC-MS/MS	17	24		α-PVP < LOQ	[26]
a-PVT	MeOH/ HCL 0.1 M at 40 °C, pulverization	LC-MS/MS	10		-/100		[21]
Benzedrone	MeOH/ACN/aq. HCOONH_4_ at 40 °C	LC-MS/MS	45	80		benzedrone < LOQ 150	[26]
Buphedrone	SPE/Bond Elute Certify I	LC-MS/MS	1	5			[24]
	M3® reagent at 100 °C	LC-MS/MS	2	20			[29]
	MeOH at 55 °C	LC-MS/MS	4.2	8.4			[20]
	MeOH/ACN/aq. HCOONH_4_ at 40 °C	LC-MS/MS	23	40			[26]
Bupropion	MeOH/ACN/aq. HCOONH_4_ at 40 °C	LC-MS/MS	17	18			[26]
Buthylone	M3® reagent at 100 °C	LC-MS/MS	2	20			[29]
Cathinone	MeOH/ HCL 0.25 M at 50 °C	GC/MS	3	20			[19]
	MeOH/ HCL 0.1 M at 40 °C, pulverization	LC-MS/MS	10		-/100		[21]
	MeOH/ACN/aq. HCOONH_4_ at 40 °C	LC-MS/MS	23	55		100−1,270 390 (pubic hair)	[26]
Dibutylone	MeOH/ACN/aq. HCOONH_4_ at 40 °C	LC-MS/MS	14	23			[26]
Diethylcathinone	M3® reagent at 100 °C	LC-MS/MS	2	20			[29]
Dimethylcathinone	M3® reagent at 100 °C	LC-MS/MS	2	20			[29]
Ethcathinone	M3® reagent at 100 °C	LC-MS/MS	2	20			[29]
	SPE/Bond Elute Certify I	LC–MS/MS	2.3	5		11.0/ 0−2.5 cm	[24]
	MeOH at 55 °C	LC-MS/MS	3.1	6.2			[20]
	0.1 M HCOOH at 45 °C	LC-MS/MS	20	20			[25]
Ethylone	SPE/Bond Elute Certify I	LC-MS/MS	0.1	1			[24]
	0.1 M HCOOH at 45 °C	LC-MS/MS	2	5			[25]
	M3® reagent at 100 °C	LC-MS/MS	2	20			[29]
	MeOH/ACN/aq. HCOONH_4_ at 40 °C	LC-MS/MS	7	12			[26]
	MeOH, MeOH/HCL 33 %	LC-MS/MS	10				[27]
	MeOH/ HCL 0.1 M at 40 °C, pulverization	LC-MS/MS	10		-/100		[21]
Eutylone	MeOH/ACN/aq. HCOONH_4_ at 40 °C	LC-MS/MS	16	23			[26]
Heliomethylamine	MeOH/ACN/aq. HCOONH_4_ at 40 °C	LC-MS/MS	7	8			[26]
MDBC	MeOH/ HCL 0.1 M at 40 °C, pulverization	LC-MS/MS	10		-/100		[21]
MDMC	MSPE: MeOH/ HCL 0.1 M at 60 °C, SPE/ C18	LC-MS/MS	1	2			[33]
	0.1 M HCOOH at 45 °C	LC-MS/MS	2	20			[25]
	M3® reagent at 100 °C	LC-MS/MS	2	20			[29]
	MeOH at 55 °C	LC-MS/MS	3.2	6.4		28	[20]
	MeOH, MeOH/HCL 33 %	LC-MS/MS	10				[27]
	MeOH/ HCL 0.1 M at 40 °C, pulverization	LC-MS/MS	10		-/100		[21]
	MeOH/ACN/aq. HCOONH_4_ at 40 °C	LC-MS/MS	12	34			[26]
MDPPP	MeOH/ACN/aq. HCOONH_4_ at 40 °C	LC-MS/MS	7	22			[26]
	MeOH/ HCL 0.1 M at 40 °C, pulverization	LC-MS/MS	10		-/100		[21]
MDPV	Basic digestion, SPE/Extrelut column	LC-MS	0.02 ng/ 10-mm	0.05 ng/10-mm		16−22 ng/10-mm	[35]
	MSPE: MeOH/ HCL 0.1 M at 60 °C, SPE/ C18	LC-MS/MS	0.2	2			[33]
	Acid/basic digestion at 95 °C, LLE with hexane/EA	LC-MS/MS	0.5	LLOQ: 1		1000	[36]
	Acid digestion at 45 °C, PLE, SPE/C18 cartridge	LC-HRMS	0.5	8			[23]
	SPE/Bond Elute Certify I	LC-MS/MS	0.5	1			[24]
	Incubation at 95 °C, LLE with Hept/EA, DCM/Isopropanol	LC-HRMS- Orbitrap	1		5/-		[30]
	MeOH/ HCL 0.1 M at 40 °C, pulverization	LC-MS/MS	1		-/100		[21]
	0.1 M HCOOH at 45 °C	LC-MS/MS	2	5		50	[25]
	MeOH at 55 °C	LC-MS/MS	2	4			[20]
	M3® reagent at 100 °C	LC-MS/MS	2	20			[29]
	MeOH, MeOH/HCL 33%	LC-MS/MS	10				[27]
	MeOH/ACN/aq. HCOONH_4_ at 40 °C	LC-MS/MS	10	23		20−800	[26]
Mephtetramine	ACN/H_2_0/ TFA at 40 °C	LC–HRMS-Orbitrap	50	200			[37]
Metamfepramone	MeOH/ACN/aq. HCOONH_4_ at 40 °C	LC-MS/MS	7	10		metamfepramone < LOQ 10	[26]
Methcathinone or ephedrone (MC)	SPE/Bond Elute Certify I	LC-MS/MS	1	5		1600.0/ 0−2.5 cm695.6/ 2.5 −5 cm	[24]
	M3® reagent at 100 °C	LC-MS/MS	2	20			[29]
	MeOH, MeOH/HCL 33 %	LC-MS/MS	10				[27]
	MeOH/ HCL 0.1 M at 40 °C, pulverization	LC-MS/MS	10		-/100		[21]
	MeOH/ HCL 0.25 M at 50 °C	GC/MS	11	40			[19]
	MeOH/ACN/aq. HCOONH_4_ at 40 °C	LC-MS/MS	15	29			[26]
Methylbuphedrone	MeOH/ACN/aq. HCOONH_4_ at 40 °C	LC-MS/MS	15	46			[26]
MOPPP	MeOH/ HCL 0.1 M at 40 °C, pulverization	LC-MS/MS	1		-/100		[21]
	MeOH/ACN/aq. HCOONH_4_ at 40 °C	LC-MS/MS	6	18		10	[26]
MPBP	MeOH/ HCL 0.1 M at 40 °C, pulverization	LC-MS/MS	1		-/100		[21]
	MeOH/ACN/aq. HCOONH_4_ at 40 °C	LC-MS/MS	9	23			[26]
Naphyrone or naphthylpyrovalerone (NPV)	MeOH/ HCL 0.1 M at 40 °C, pulverization	LC-MS/MS	1		-/100		[21]
	M3® reagent at 100 °C	LC-MS/MS	2	20			[29]
	SPE/Bond Elute Certify I	LC-MS/MS	2.5	5			[24]
	MeOH/ACN/aq. HCOONH_4_ at 40 °C	LC-MS/MS	6	18			[26]
	0.1 M HCOOH at 45 °C	LC-MS/MS	10	20			[25]
N-ethylcathinone (EC)	MeOH/ HCL 0.1 M at 40 °C, pulverization	LC-MS/MS	10		-/100		[21]
	MeOH/ACN/aq. HCOONH_4_ at 40 °C	LC-MS/MS	16	44			[26]
N,N-DMC	MeOH/ HCL 0.1 M at 40 °C, pulverization	LC-MS/MS	10		-/100		[21]
Penthedrone	M3® reagent at 100 °C	LC-MS/MS	2	20			[29]
Pentedrone	SPE/Bond Elute Certify I	LC-MS/MS	0.4	1		198.4/ 0−2.5 cm586.2/ 2.5 −5 cm	[24]
	0.1 M HCOOH at 45 °C	LC-MS/MS	2	20			[25]
	MeOH at 55 °C	LC-MS/MS	3.9	7.8			[20]
	MeOH/ HCL 0.1 M at 40 °C, pulverization	LC-MS/MS	10		-/100		[21]
	MeOH/ACN/aq. HCOONH_4_ at 40 °C	LC-MS/MS	18	39		7340	[26]
Penthylone	M3® reagent at 100 °C	LC-MS/MS	2	20			[29]
Pentylone	SPE/Bond Elute Certify I	LC-MS/MS	0.1	1			[24]
	0.1 M HCOOH at 45 °C	LC-MS/MS	2	20			[25]
	MeOH/ACN/aq. HCOONH_4_ at 40 °C	LC-MS/MS	8	23			[26]
	MeOH/ HCL 0.1 M at 40 °C, pulverization	LC-MS/MS	10		-/100		[21]
PMMC	SPE/Bond Elute Certify I	LC-MS/MS	0.2	1			[24]
	MSPE: MeOH/ HCL 0.1 M at 60 °C, SPE/ C18	LC-MS/MS	1	2			[33]
	0.1 M HCOOH at 45 °C	LC-MS/MS	2	20			[25]
	MeOH/ACN/aq. HCOONH_4_ at 40 °C	LC-MS/MS	4	18			[26]
	MeOH/ HCL 0.1 M at 40 °C, pulverization	LC-MS/MS	10		-/100		[21]
Pyrovalerone	MeOH/ HCL 0.1 M at 40 °C, pulverization	LC-MS/MS	1		-/100		[21]
	MeOH/ACN/aq. HCOONH_4_ at 40 °C	LC-MS/MS	8	14			[26]
2-FMC	Acid digestion at 45 °C, PLE, SPE/C18 cartridge	LC-HRMS	4	50			[23]
2-Methoxymethcathinone	Acid digestion at 45 °C, PLE, SPE/C18 cartridge	LC-HRMS	7	20			[23]
3,4-DMMC	SPE/Bond Elute Certify I	LC-MS/MS	0.3	1		2800.0/ 0−2.5 cm572.6/ 2.5 −5 cm	[24]
	M3® reagent at 100 °C	LC-MS/MS	2	20			[29]
	0.1 M HCOOH at 45 °C	LC-MS/MS	5	20			[25]
	MeOH/ HCL 0.1 M at 40 °C, pulverization	LC-MS/MS	10		-/100		[21]
	MeOH/ACN/aq. HCOONH_4_ at 40 °C	LC-MS/MS	17	43			[26]
3-FMC	MeOH/ HCL 0.1 M at 40 °C, pulverization	LC-MS/MS	10		-/100		[21]
	MeOH/ACN/aq. HCOONH_4_ at 40 °C	LC-MS/MS	31	35			[26]
3-MMC	MeOH/ HCL 0.1 M at 40 °C, pulverization	LC-MS/MS	10		-/100		[21]
	MeOH/ TFA at 45 °C, after pulver.	LC-HRMS-Orbitrap	20	100		25.800	[38]
4-BMC	MeOH/ACN/aq. HCOONH_4_ at 40 °C	LC-MS/MS	52	95		2730	[26]
4-FMC	SPE/Bond Elute Certify I	LC-MS/MS	1	5		41.1/ 0−2.5 cm45.6/ 2.5 −5 cm	[24]
	M3® reagent at 100 °C	LC-MS/MS	2	20			[29]
	0.1 M HCOOH at 45 °C	LC-MS/MS	5	10			[25]
	MSPE: MeOH/ HCL 0.1 M at 60 °C, SPE/ C18	LC-MS/MS	5	10			[33]
	MeOH/ HCL 0.1 M at 40 °C, pulverization	LC-MS/MS	10		-/100		[21]
4-FPP	Acid digestion at 45 °C, PLE, SPE/C18 cartridge	LC-HRMS	7	30			[23]
4-MBu	MeOH/ HCL 0.1 M at 40 °C, pulverization	LC-MS/MS	10		-/100		[21]
4-MEC	SPE/Bond Elute Certify I	LC-MS/MS	0.4	1		2200.0/ 0−2.5 cm591.0/ 2.5 −5 cm	[24]
	Acid/basic digestion at 95 °C, LLE with hexane/EA	LC-MS/MS	0.5	LLOQ: 1		30,000	[36]
	M3® reagent at 100 °C	LC-MS/MS	2	20			[29]
	MeOH at 55 °C	LC-MS/MS	3	6			[20]
	0.1 M HCOOH at 45 °C	LC-MS/MS	5	20		< LOQ; 26	[25]
	MeOH/ HCL 0.1 M at 40 °C, pulverization	LC-MS/MS	10		-/100		[21]
	MeOH/ACN/aq. HCOONH_4_ at 40 °C	LC-MS/MS	11	33			[26]
4-MMC	MSPE: MeOH/ HCL 0.1 M at 60 °C, SPE/ C18	LC-MS/MS	1	2			[33]
	SPE/Bond Elute Certify I	LC-MS/MS	1	5		6200.0/ 0−2.5 cm1500.0/ 2.5 −5 cm	[24]
	0.1 M HCOOH at 45 °C	LC-MS/MS	2	20			[25]
	M3® reagent at 100 °C	LC-MS/MS	2	20			[29]
	MeOH at 55 °C	LC-MS/MS	2.4	4.8		50−59	[20]
	Enzymatic digestion, LLE with chloroform/EtOH/DEE	LC-MS/MS	2.5	5		21.11	[39]
	Acid digestion at 45 °C, PLE, SPE/C18 cartridge	LC-HRMS	4	10			[23]
	Incubation at 95 °C, LLE with Hept/EA, DCM/Isopropanol	LC-HRMS- Orbitrap	5		50/-		[30]
	MeOH/ACN/aq. HCOONH_4_ at 40 °C	LC-MS/MS	7	10		220−3.500 mephedrone < LOQ	[26]
	MeOH, MeOH/HCL 33 %	LC-MS/MS	10				[27]
	MeOH/ HCL 0.1 M at 40 °C, pulverization	LC-MS/MS	10		-/100		[21]
	Neutral digestion at 40 °C, LLE with EA	GC-MS	80	200		200−313,20	[40]
4-Methylnorephedrine	Enzymatic digestion, LLE with chloroform/EtOH/DEE	LC-MS/MS	5	10			[39]

As expected, methanol, alone or mixed with other organic solvents or aqueous hydrochloric solutions, was the most used solvent for cathinones extraction from hair, since they are holding dissociation constants in the basic range [66]. The most effective extraction mixture seems to be MeOH/ACN/H_2_O plus ammonium formate, and acidified methanol (0.1 M) were used to extract 31 [26] and 28 [21] cathinones, respectively.

Remarkably, the LOD of 3-MMC in pubic hair after extraction with methanol: TFA, and LC-HRMS-Orbitrap analysis [38] was higher than the respective LODs achieved by acidified MeOH and LC–MS/MS analysis [21]. Generally, SPE and LLE methods were proved to be equally effective by different low or high resolution LC–MS methods. As expected, higher LOD was achieved during GC–MS analysis [40] for 4-MMC, compared to that obtained with different extraction methods and LC–MS/MS analysis and detection [20,21,[24], [25], [26], [27],^29^,^33^,^39^].

### 2.3 Determination of synthetic cannabinoids in hair

Synthetic cannabinoids (SCs) are among the most popular NPS that display high-affinity binding to the CB1 and CB2 cannabinoid receptors and demonstrate a pharmacological profile like trans-Δ9 -tetrahydrocannabinol (THC) [1,67]. They hold hallucinogenic, hypnotic, and/ or sedative effects. Seventeen studies reported the presence of 109 synthetic cannabinoids in hair, using LC–MS/MS [21,22,25,29,[41], [42], [43], [44], [45],^47^,^48^,^50^,^51^], GC/MS [46], LC-HRMS [23,30]. The relative data are presented in Table 3. Seven of these studies [[21], [22], [23],^25^,^29^,^30^,^46^] have been engaged with the simultaneous analysis of cannabinoids with other NPS classes.

**Table 3:** Selected parameters of hair analysis for Synthetic Cannabinoids.

**NPS**	**Extraction Method**	**Method of Analysis**	**LOD (pg/mg)**	**LOQ (pg/mg)**	**LOI/ LOR (pg/mg)**	**Concentrations- Clinical/forensic samples (pg/mg)**	**References**
**CANNABINOIDS: Benzoylindoles**							
AM-2233	MeOH/ HCL 0.1 M at 40 °C, pulverization	LC-MS/MS	1		-/100		[21]
	M3® reagent at 100 °C	LC-MS/MS	5	25			[29]
	MeOH at 45 °C	LC-MS/MS	10	20			[25]
AM-694	EtOH	LC-MS/MS	0.5	0.5			[41]
	Basic digestion at 95 °C, LLE with hexane/EA	LC-MS/MS	0.8	2.6			[42]
	MeOH/ HCL 0.1 M at 40 °C, pulverization	LC-MS/MS	1		-/100		[21]
	M3® reagent at 100 °C	LC-MS/MS	5	25			[29]
	MeOH at 45 °C	LC-MS/MS	10	20		30	[25]
	Enzymatic Digestion, LLE neutral and basic with DEE/EA	LC-MS/MS	20				[22]
RCS-4	EtOH	LC-MS/MS	0.5	0.5			[41]
	Basic digestion at 95 °C, LLE with hexane/EA	LC-MS/MS	0.7	2.3			[42]
	MeOH/ HCL 0.1 M at 40 °C, pulverization	LC-MS/MS	1		-/100		[21]
	M3® reagent at 100 °C	LC-MS/MS	5	25			[29]
	MeOH at 45 °C	LC-MS/MS	10	20			[25]
	Enzymatic Digestion, LLE neutral and basic with DEE/EA	LC-MS/MS	100				[22]
RCS4−2-methoxy	MeOH/ HCL 0.1 M at 40 °C, pulverization	LC-MS/MS	1		-/100		[21]
RCS4-C-4	MeOH/ HCL 0.1 M at 40 °C, pulverization	LC-MS/MS	1		-/100		[21]
RCS-4 ortho isomer	EtOH	LC-MS/MS	0.5	0.5			[41]
WIN 48.098	EtOH	LC-MS/MS	0.5	0.5			[41]
	Basic digestion at 95 °C, LLE with hexane/EA	LC-MS/MS	0.7	2.3			[42]
	MeOH/ HCL 0.1 M at 40 °C, pulverization	LC-MS/MS	1		-/100		[21]
	MeOH at 45 °C	LC-MS/MS	5	20			[25]
**Naphthoylindoles**							
AKB-48	MeOH at 38 °C	LC-MS/MS	Range 0.1 to 10	Range 0.1–20			[43]
AM-1220 azepane	MeOH/ HCL 0.1 M at 40 °C, pulverization	LC-MS/MS	10		-/100		[21]
AM-1220	Basic digestion at 95 °C, LLE with hexane/EA	LC-MS/MS	0.4	1.3		1,3	[42]
	MeOH/ HCL 0.1 M at 40 °C, pulverization	LC-MS/MS	10		-/100		[21]
AM-1241	MeOH/ HCL 0.1 M at 40 °C, pulverization	LC-MS/MS	10		-/100		[21]
AM-1248	MeOH/ HCL 0.1 M at 40 °C, pulverization	LC-MS/MS	1		-/100		[21]
AM-2201	MeOH at 38 °C	LC-MS/MS	0.05	0.1		1.7−739,0	[44]
	Acid digestion at 95 °C, LLE with hexane/EA	LC-MS/MS	0.35	1			[45]
	Basic digestion at 95 °C, LLE with hexane/EA	LC-MS/MS	0.7	2.3			[42]
	EtOH	LC-MS/MS	0.5	0.5			[41]
	M3® reagent at 100 °C	LC-MS/MS	1	10			[29]
	MeOH at 45 °C	LC-MS/MS	10	10			[25]
	Enzymatic Digestion, LLE neutral and basic with DEE/EA	LC-MS/MS	20				[22]
	Basic digestion at 90 °C, LLE with hexane/EA	GC/MS	1000	1000		5516	[46]
AM-2201 N-4-OH M	MeOH at 38 °C	LC-MS/MS	0.05	0.1		0.4	[44]
AM-2232	MeOH/ HCL 0.1 M at 40 °C, pulverization	LC-MS/MS	10		-/100		[21]
PX-1 (derivative of AM2201)	M3® reagent at 100 °C	LC-MS/MS	5	25			[29]
AM-2201 N-6-OHindole M	MeOH at 38 °C	LC-MS/MS	0.05	0.1		0,2−3,1	[44]
BB-22 (analog of JWH 018)	MeOH/ HCL 0.1 M at 40 °C, pulverization	LC-MS/MS	1		-/100		[21]
	Acid digestion at 95 °C, LLE with hexane/EA	LC-MS/MS	3	10			[45]
EAM-2201	Acid digestion at 95 °C, LLE with hexane/EA	LC-MS/MS	0.35	1			[45]
JWH-007	Basic digestion at 95 °C, LLE with hexane/EA	LC-MS/MS	0.2	0.7			[42]
	EtOH	LC-MS/MS	0.5	0.5			[41]
	MeOH/ HCL 0.1 M at 40 °C, pulverization	LC-MS/MS	1		-/100		[21]
	M3® reagent at 100 °C	LC-MS/MS	3	25			[29]
	MeOH at 45 °C	LC-MS/MS	5	10			[25]
JWH-015	EtOH	LC-MS/MS	0.5	0.5			[41]
	Basic digestion at 95 °C, LLE with hexane/EA	LC-MS/MS	0.6	2			[42]
	MeOH/ HCL 0.1 M at 40 °C, pulverization	LC–MS/MS	1		-/100		[21]
	MeOH at 45 °C	LC-MS/MS	2	10			[25]
	Enzymatic Digestion, LLE neutral and basic with DEE/EA	LC-MS/MS	500				[22]
JWH-018	MeOH at 38 °C	LC-MS-MS	0.05	0.1		10−1700	[47]
	Basic digestion at 95 °C, LLE with hexane/EA	LC-MS/MS	0.18	0.59		0,6−70,5	[48]
	Acid digestion at 95 °C, LLE with hexane/EA	LC-MS/MS	0.35	1			[45]
	EtOH	LC-MS/MS	0.5	0.5		5.1−5.7	[41]
	Basic digestion at 95 °C, LLE with hexane/EA	LC-MS/MS	0.9	3		3.1−17.3	[42]
	MeOH/ HCL 0.1 M at 40 °C, pulverization	LC–MS/MS	1		-/100		[21]
	M3® reagent at 100 °C	LC-MS/MS	3	25			[29]
	Incubation at 95 °C, LLE with Hept/EA, DCM/Isopropanol	LC-HRMS- Orbitrap	5		50/-	0.8–70.5	[30]
	MeOH at 45 °C	LC-MS/MS	5	10		Case 1: 20, Case 2: 90 Case 3: traces below LOQ	[25]
	Enzymatic Digestion, LLE neutral and basic with DEE/EA	LC-MS/MS	500				[22]
	MeOH at 38 °C	LC-MS/MS				0.4−59.2	[44]
JWH-018 N-COOH M	MeOH at 38 °C	LC-MS-MS	0.05	0.1			[47]
	MeOH at 38 °C	LC-MS/MS				0.2−1.1	[44]
JWH-018 N-4-OH M	MeOH at 38 °C	LC-MS-MS	0.05	0.1			[47]
JWH-018 N-(5-OH) M	MeOH at 38 °C	LC-MS-MS	0.05	0.1		3−85	[47]
	Acid digestion at 95 °C, LLE with hexane/EA	UPLC-MS/MS	0.35	1			[45]
	Acid digestion at 45 °C, PLE, SPE/C18 cartridge	LC-HRMS	7	30			[23]
	MeOH at 38 °C	LC-MS/MS				0.3−37.2	[44]
JWH-018 adamantyl	MeOH/ HCL 0.1 M at 40 °C, pulverization	LC–MS/MS	1		-/100		[21]
THJ 018 (analog of JWH 018)	M3® reagent at 100 °C	LC-MS/MS	3	25			[29]
JWH-019	Acid digestion at 95 °C, LLE with hexane/EA	LC-MS/MS	0.35	1			[45]
	EtOH	LC-MS/MS	0.5	0.5			[41]
	MeOH/ HCL 0.1 M at 40 °C, pulverization	LC-MS/MS	1		-/100		[21]
	Basic digestion at 95 °C, LLE with hexane/EA	LC-MS/MS	1	3.3		3.8−4.1	[42]
	M3® reagent at 100 °C	LC-MS/MS	3	25			[29]
	MeOH at 45 °C	LC-MS/MS	5	20			[25]
	Basic digestion at 90 °C, LLE with hexane/EA	GC/MS	50	100		4996	[46]
JWH-073	MeOH at 38 °C	LC-MS-MS	0.05	0.1		2−55	[47]
	Basic digestion at 95 °C, LLE with hexane/EA	LC-MS/MS	0.1	0.33		0.5−413.3	[48]
	Acid digestion at 95 °C, LLE with hexane/EA	LC-MS/MS	0.35	1			[45]
	EtOH	LC-MS/MS	0.5	0.5		0.7−3.2	[41]
	Basic digestion at 95 °C, LLE with hexane/EA	LC-MS/MS	0.5	1.6		1.6−50.5	[42]
	MeOH/ HCL 0.1 M at 40 °C, pulverization	LC-MS/MS	10		-/100		[21]
	MeOH at 45 °C	LC-MS/MS	10	20		Case 1: below LOQ, Case 2: 2.100 Case 3: traces below LOQ	[25]
	Enzymatic Digestion, LLE neutral and basic with DEE/EA	LC-MS/MS	500				[22]
	MeOH at 38 °C	LC-MS/MS				0.1−0.8	[44]
JWH-073−4-N(OHbutyl)	Acid digestion at 95 °C, LLE with hexane/EA	LC-MS/MS	0.35	1			[45]
JWH-073 N-3-OH M	MeOH at 38 °C	LC-MS-MS	0.05	0.1			[47]
JWH-073 N-COOH M	MeOH at 38 °C	LC-MS-MS	0.05	0.1			[47]
	MeOH at 38 °C	LC-MS/MS				0.3	[44]
JWH-073 N-4-OH M	MeOH at 38 °C	LC-MS-MS	0.1	0.1			[47]
JWH073 4-methylnapthyl	MeOH/ HCL 0.1 M at 40 °C, pulverization	LC-MS/MS	1		-/100		[21]
JWH073 N-(3-methylbutyl)	MeOH/ HCL 0.1 M at 40 °C, pulverization	LC-MS/MS	1		-/100		[21]
JWH-081	Incubation at 45 °C, SPE/MCX® Oasis cartridge	LC-MS/MS	0.5	0.5		1st segment: 78 3rd segment: 1100	[31]
	Basic digestion at 95 °C, LLE with hexane/EA	LC-MS/MS	0.6	2		8.0−194	[42]
	MeOH/ HCL 0.1 M at 40 °C, pulverization	LC-MS/MS	1		-/100		[21]
	Acid digestion at 45 °C, PLE, SPE/C18 cartridge	LC-HRMS	3	10			[23]
	M3® reagent at 100 °C	LC-MS/MS	3	25			[29]
	MeOH at 45 °C	LC-MS/MS	5	20		Case 1: 470 Case 3: traces below LOQ	[25]
	Basic digestion at 90 °C, LLE with hexane/EA	GC/MS	100	100		5.533	[46]
JWH-098	M3® reagent at 100 °C	LC-MS/MS	3	25			[29]
	MeOH at 45 °C	LC-MS/MS	5	20			[25]
JWH-122	MeOH at 38 °C	LC-MS/MS	0.05	0.1		0.1- 402.0	[44]
	Acid digestion at 95 °C, LLE with hexane/EA	LC-MS/MS	0.35	1		200/ 0−2 cm450/ 2−4 cm430/4 −6 cm	[45]
	EtOH	LC-MS/MS	0.5	0.5			[41]
	Basic digestion at 95 °C, LLE with hexane/EA	LC-MS/MS	0.9	3		7,4−2,800	[42]
	MeOH/ HCL 0.1 M at 40 °C, pulverization	LC-MS/MS	1		-/100		[21]
	M3® reagent at 100 °C	LC-MS/MS	3	20			[29]
	MeOH at 45 °C	LC-MS/MS	10	20			[25]
	Basic digestion at 90 °C, LLE with hexane/EA	GC/MS	100	100		5366	[46]
	Enzymatic Digestion, LLE neutral and basic with DEE/EA	LC-MS/MS	500				[22]
JWH-122 N-(4-pentenyl)	MeOH/ HCL 0.1 M at 40 °C, pulverization	LC-MS/MS	1		-/100		[21]
JWH-122 N-5-OH	MeOH at 38 °C	LC-MS/MS	0.05	0.1		0.1- 3.5	[44]
JWH-200	Basic digestion at 95 °C, LLE with hexane/EA	LC-MS/MS	0.02	0.07			[48]
	Acid digestion at 95 °C, LLE with hexane/EA	LC-MS/MS	0.35	1			[45]
	Basic digestion at 95 °C, LLE with hexane/EA	LC-MS/MS	0.4	1.3			[42]
	EtOH	LC-MS/MS	0.5				[41]
	MeOH at 45 °C	LC-MS/MS	5	10			[25]
	MeOH/ HCL 0.1 M at 40 °C, pulverization	LC-MS/MS	1		-/100		[21]
	Acid digestion at 45 °C, PLE, SPE/C18 cartridge	LC-HRMS	1	5			[23]
	Enzymatic Digestion, LLE neutral and basic with DEE/EA	LC-MS/MS	20				[22]
JWH-210	MeOH at 38 °C	LC-MS/MS	Range 0.1 to 10	Range 0.1–20		0.06−7.6	[43]
	EtOH	LC-MS/MS	0.5	0.5		0.5−5.2	[41]
	Basic digestion at 95 °C, LLE with hexane/EA	LC-MS/MS	0.7	2.3		2.3−5.1	[42]
	MeOH/ HCL 0.1 M at 40 °C, pulverization	LC–MS/MS	1		-/100		[21]
	M3® reagent at 100 °C	LC-MS/MS	3	25			[29]
	Enzymatic Digestion, LLE neutral and basic with DEE/EA	LC-MS/MS	10				[22]
JWH-398	Basic digestion at 95 °C, LLE with hexane/EA	LC-MS/MS	0.3	1			[42]
	MeOH/ HCL 0.1 M at 40 °C, pulverization	LC–MS/MS	1		-/100		[21]
	M3® reagent at 100 °C	LC-MS/MS	3	25			[29]
	EtOH	LC-MS/MS	5	5			[41]
	MeOH at 45 °C	LC-MS/MS	5	10			[25]
MAM-2201	MeOH/ HCL 0.1 M at 40 °C, pulverization	LC-MS/MS	1		-/100		[21]
	MeOH at 38 °C	LC-MS/MS	0.05	0.1		0.2−276.0	[44]
MAM-2201 N-4-OH M	MeOH at 38 °C	LC-MS/MS	0.05	0.1			[44]
MAM 2201 N-(5-pentanoic acid) -potential phase 1 metabolite of JWH 122	MeOH at 38 °C	LC-MS/MS	0.05	0.1			[44]
	Acid digestion at 45 °C, PLE, SPE/C18 cartridge	LC-HRMS	5	40			[23]
WIN 55, 212−2	EtOH	LC-MS/MS	0.5	0.5			[41]
	Basic digestion at 95 °C, LLE with hexane/EA	LC-MS/MS	0.8	2.6			[42]
	MeOH/ HCL 0.1 M at 40 °C, pulverization	LC–MS/MS	1		-/100		[21]
	Acid digestion at 45 °C, PLE, SPE/C18 cartridge	LC-HRMS	8	30			[23]
5 F-AKB48	MeOH at 38 °C	LC-MS/MS	Range 0.1 to 10	Range 0.1–20			[43]
	Acid digestion at 95 °C, LLE with hexane/EA	LC-MS/MS	3	10			[45]
	M3® reagent at 100 °C	LC-MS/MS	5	30			[29]
5 F NNEI-2 (analog of JWH 018)	M3® reagent at 100 °C	LC-MS/MS	5	30			[29]
**Phenylacetylindoles**							
JWH-203	EtOH	LC-MS/MS	0.5	0.5			[41]
	Basic digestion at 95 °C, LLE with hexane/EA	LC-MS/MS	0.7	2.3			[42]
	MeOH/ HCL 0.1 M at 40 °C, pulverization	LC–MS/MS	1		-/100		[21]
	M3® reagent at 100 °C	LC-MS/MS	3	25			[29]
	Enzymatic Digestion, LLE neutral and basic with DEE/EA	LC-MS/MS	50				[22]
JWH-250	Basic digestion at 95 °C, LLE with hexane/EA	LC-MS/MS	0.04	0.13		1.5−729.4	[48]
	Acid digestion at 95 °C, LLE with hexane/EA	LC-MS/MS	0.35	1			[45]
	Basic digestion at 95 °C, LLE with hexane/EA	LC-MS/MS	0.5	1.6		4.8−83.4	[42]
	EtOH	LC-MS/MS	0.5	0.5		0.5−24	[41]
	Acid digestion at 45 °C, PLE, SPE/C18 cartridge	LC-HRMS	1	9			[23]
	MeOH/ HCL 0.1 M at 40 °C, pulverization	LC–MS/MS	1		-/100		[21]
	Enzymatic Digestion, LLE neutral and basic with DEE/EA	LC-MS/MS	10				[22]
	MeOH at 45 °C	LC-MS/MS	10	10			[25]
	Basic digestion at 90 °C, LLE with hexane/EA	GC/MS	50	100		5.320	[46]
JWH-251	MeOH at 45 °C	LC-MS/MS	10	20			[25]
	EtOH	LC-MS/MS	0.5	0.5			[41]
	MeOH/ HCL 0.1 M at 40 °C, pulverization	LC-MS/MS	1		-/100		[21]
	Basic digestion at 95 °C, LLE with hexane/EA	LC-MS/MS	0.3	1			[42]
	M3® reagent at 100 °C	LC-MS/MS	3	25			[29]
JWH-302	M3® reagent at 100 °C	LC-MS/MS	2	25			[29]
RCS-8	EtOH	LC-MS/MS	0.5	0.5			[41]
	Basic digestion at 95 °C, LLE with hexane/EA	LC-MS/MS	0.9	3			[42]
	MeOH/ HCL 0.1 M at 40 °C, pulverization	LC-MS/MS	1		-/100		[21]
	M3® reagent at 100 °C	LC-MS/MS	5	30			[29]
	MeOH at 45 °C	LC-MS/MS	5	10			[25]
**Naphthoylpyrroles**							
JWH-030	MeOH/ HCL 0.1 M at 40 °C, pulverization	LC-MS/MS	1		-/100		[21]
	MeOH at 45 °C	LC-MS/MS	10	10			[25]
JWH-147	M3® reagent at 100 °C	LC-MS/MS	3	25			[29]
	MeOH at 45 °C	LC-MS/MS	10	20			[25]
JWH-307	MeOH/ HCL 0.1 M at 40 °C, pulverization	LC-MS/MS	1		-/100		[21]
	Basic digestion at 95 °C, LLE with hexane/EA	LC-MS/MS	1.3	4.3			[42]
	MeOH at 45 °C	LC-MS/MS	10	20			[25]
**OTHER CANNABINOIDS**							
AB-CHMINACA	MeOH	LC-MS/MS	0.1	LLOQ: 2.5		∼40−1850	[49]
	MeOH at 38 °C	LC-MS/MS	0.5	2		2.2−1512.0	[50]
	MeOH at 38 °C	LC-MS/MS	Range 0.1 to 10	Range 0.1–20		2.5−15300.0	[43]
	M3® reagent at 100 °C	LC-MS/MS	5	25			[29]
AB-CHMINACA M1A	MeOH at 38 °C	LC-MS/MS	Range 0.1 to 10	Range 0.1–20		18.3 (1 case)	[43]
AB-CHMINACA M2	MeOH at 38 °C	LC-MS/MS	1	5			[50]
	MeOH at 38 °C	LC-MS/MS	Range 0.1 to 10	Range 0.1 to 20		0.5−35.1	[43]
AB-CHMINACA M3A	MeOH at 38 °C	LC-MS/MS	2.5	5			[50]
AB-CHMINACA M4	MeOH at 38 °C	LC-MS/MS	Range 0.1 to 10	Range 0.1–20		59.8	[43]
	MeOH at 38 °C	LC-MS/MS	2.5	5			[50]
AB-CHMINACA M5A	MeOH at 38 °C	LC-MS/MS	10	50			[50]
AB-CHMINACA M6	MeOH at 38 °C	LC-MS/MS	2.5	5			[50]
AB-CHMINACA M7	MeOH at 38 °C	LC-MS/MS	2.5	10			[50]
AB-CHMINACA Valine (METABOLITE)	MeOH	LC-MS/MS	0.1			∼100−450	[49]
AB-FUBINACA	MeOH at 38 °C	LC-MS/MS	Range 0.1 to 10	Range 0.1–20			[43]
	Acid digestion at 95 °C, LLE with hexane/EA	LC-MS/MS	3	10			[45]
	M3® reagent at 100 °C	LC-MS/MS	5	25			[29]
	MeOH/ HCL 0.1 M at 40 °C, pulverization	LC-MS/MS	10		-/100		[21]
AB PINACA	MeOH at 38 °C	LC-MS/MS	Range 0.1 to 10	Range 0.1–20			[43]
	MeOH/ HCL 0.1 M at 40 °C, pulverization	LC-MS/MS	10		-/100		[21]
ADB FUBINACA	M3® reagent at 100 °C	LC-MS/MS	5	25			[29]
	MeOH/ HCL 0.1 M at 40 °C, pulverization	LC-MS/MS	10		-/100		[21]
ADB-PINACA	Acid digestion at 95 °C, LLE with hexane/EA	LC-MS/MS	0.35	1			[45]
APICA	MeOH/ HCL 0.1 M at 40 °C, pulverization	LC-MS/MS	1		-/100		[21]
APINACA	MeOH/ HCL 0.1 M at 40 °C, pulverization	LC-MS/MS	1		-/100		[21]
APP FUBINACA (analog of AB-FUBINACA)	M3® reagent at 100 °C	LC-MS/MS	5	25		50	[29]
A-834,735	MeOH/ HCL 0.1 M at 40 °C, pulverization	LC-MS/MS	1		-/100		[21]
CB-13	MeOH/ HCL 0.1 M at 40 °C, pulverization	LC-MS/MS	1		-/100		[21]
	M3® reagent at 100 °C	LC-MS/MS	5	30			[29]
	MeOH at 45 °C	LC-MS/MS	10	20			[25]
CP47, 497-C8	M3® reagent at 100 °C	LC-MS/MS	5	30			[29]
	Basic digestion at 90 °C, LLE with hexane/EA	GC/MS	50	500		5.300	[46]
CUMYL 5 F PINACA	M3® reagent at 100 °C	LC-MS/MS	5	25			[29]
CUMYL-PEGACLONE	M3® reagent at 100 °C	LC-MS/MS	5	25			[29]
HU-210	MeOH/ HCL 0.1 M at 40 °C, pulverization	LC-MS/MS	1		-/100		[21]
	Basic digestion at 95 °C, LLE with hexane/EA	LC-MS/MS	3	9.9			[48]
	Basic digestion at 95 °C, LLE with hexane/EA	LC-MS/MS	24	80			[42]
STS-135	MeOH/ HCL 0.1 M at 40 °C, pulverization	LC-MS/MS	1		-/100		[21]
JWH-016	M3® reagent at 100 °C	LC-MS/MS	3	25			[29]
	MeOH at 45 °C	LC-MS/MS	5	10			[25]
JWH-020	Basic digestion at 95 °C, LLE with hexane/EA	LC-MS/MS	0.2	0.7			[42]
	EtOH	LC-MS/MS	0.5	0.5			[41]
	MeOH/ HCL 0.1 M at 40 °C, pulverization	LC-MS/MS	1		-/100		[21]
JWH-022	MeOH/ HCL 0.1 M at 40 °C, pulverization	LC-MS/MS	1		-/100		[21]
JWH-072	MeOH/ HCL 0.1 M at 40 °C, pulverization	LC-MS/MS	1		-/100		[21]
JWH-175	MeOH/ HCL 0.1 M at 40 °C, pulverization	LC-MS/MS	1		-/100		[21]
JWH-176	MeOH/ HCL 0.1 M at 40 °C, pulverization	LC-MS/MS	10		-/100		[21]
JWH-182	MeOH/ HCL 0.1 M at 40 °C, pulverization	LC-MS/MS	1		-/100		[21]
JWH-201	MeOH/ HCL 0.1 M at 40 °C, pulverization	LC-MS/MS	1		-/100		[21]
	MeOH at 45 °C	LC-MS/MS	2	10			[25]
JWH-213	MeOH/ HCL 0.1 M at 40 °C, pulverization	LC-MS/MS	1		-/100		[21]
JWH-412	MeOH/ HCL 0.1 M at 40 °C, pulverization	LC-MS/MS	1		-/100		[21]
MMB 2201	M3® reagent at 100 °C	LC-MS/MS	5	25			[29]
PB-22	MeOH at 38 °C	LC-MS/MS	Range 0.1 to 10	Range 0.1–20			[43]
	MeOH/ HCL 0.1 M at 40 °C, pulverization	LC-MS/MS	1		-/100		[21]
PB-22 5-OH-pentyl	MeOH	LC-MS/MS	0.5			∼0−450	[49]
Pravadoline	M3® reagent at 100 °C	LC-MS/MS	5	25			[29]
P X 2 (analog of 5-fluoro AB-PINACA)	M3® reagent at 100 °C	LC-MS/MS	5	25			[29]
UR-144	MeOH at 38 °C	LC-MS/MS	0.01	0.2		0.4−1.6	[51]
	MeOH/ HCL 0.1 M at 40 °C, pulverization	LC-MS/MS	1		-/100		[21]
	M3® reagent at 100 °C	LC-MS/MS	1	10		100	[29]
	Acid digestion at 45 °C, PLE, SPE/C18 cartridge	LC-HRMS	6	20			[23]
	Basic digestion at 90 °C, LLE with hexane/EA	GC/MS	50	500			[46]
UR-144 N-4-OH M	MeOH at 38 °C	LC-MS/MS	0.01	0.2		1−25.3	[51]
UR-144 N (5 Cl-pentyl)	MeOH/ HCL 0.1 M at 40 °C, pulverization	LC-MS/MS	1		-/100		[21]
UR-144 N-COOH M	MeOH at 38 °C	LC-MS/MS	0.01	0.2		0.2−7.9	[51]
UR-144 N-5-OH M	MeOH at 38 °C	LC-MS/MS	0.01	0.2		0.2−39.7	[51]
URB-754	MeOH/ HCL 0.1 M at 40 °C, pulverization	LC-MS/MS	10		-/100		[21]
XLR-11 N-4-OH M	MeOH at 38 °C	LC-MS/MS	0.2	0.2			[51]
XLR-11	MeOH at 38 °C	LC-MS/MS	0.01	0.2		0.8−5350	[51]
	Acid digestion at 45 °C, PLE, SPE/C18 cartridge	LC-HRMS	Range 0.1 to 10	Range 0.1–20			[23]
	MeOH/ HCL 0.1 M at 40 °C, pulverization	LC-MS/MS	1		-/100		[21]
5 CL AB PINACA	M3® reagent at 100 °C	LC-MS/MS	5	25			[29]
5 F-AB-PINACA	Acid digestion at 95 °C, LLE with hexane/EA	LC-MS/MS	8	25			[45]
	MeOH/ HCL 0.1 M at 40 °C, pulverization	LC-MS/MS	10		-/100		[21]
5-F ADB	M3® reagent at 100 °C	LC-MS/MS	5	25			[29]
5 F-APINACA	MeOH/ HCL 0.1 M at 40 °C, pulverization	LC-MS/MS	10		-/100		[21]
5-fluoro PB-22	MeOH/ HCL 0.1 M at 40 °C, pulverization	LC-MS/MS	1		-/100		[21]
	Acid digestion at 95 °C, LLE with hexane/EA	LC-MS/MS	3	10			[45]
	MeOH	LC-MS/MS	10	LLOQ: 1		∼200−1900	[49]
5 F-PB-22 3-carboxyindole	MeOH	LC-MS/MS	10			∼200−800	[49]

From chemical point of view, the majority of SCs molecules consist of 22–26 carbon atoms being highly lipophilic [2]. They are soluble in solvents with low polarity (e.g. isooctane) as well as in methanol, ethanol, acetonitrile, ethyl acetate, acetone and other medium polar organic solvents while their solubility in water is low [1].

**Benzoylindoles:** A total of six studies were conducted for the determination of seven benzoylindoles in hair by LC–MS methods [21,22,25,29,41,42].

The most frequent extraction method applied was acidified methanol (0.1 M HCl) [21], being the most effective compared to methanol alone [25] or with other mixtures of organic solvents [22,41,42] used for LLE of NPS from hair, except for WIN48.098 [25].

**Naphthoylindoles:** A total of 14 studies have been conducted for the determination of 42 naphthoylindoles in hair [[21], [22], [23],^25^,^29^,^30^,^41^,[43], [44], [45], [46], [47], [48]]. The principal extraction method applied is MeOH/ 0.1 M HCL, to define 22 of them [21]. Methanol [44,47] was the most efficient extraction method for most naphthoindoles than with other organic solvents, or SPE methods. However, the LODs of JWH-398, AM1220, WIN 55, 212−2, MAM-2201 N (5-pentanoic acid), and JWH-018N-(5−OH), after LLE with various solvents [41,42], were comparable to those attained with methanol extraction [25,44] or acidified methanol [21].

As expected, higher LODs were achieved during GS-MS analysis [46] for determination of AM2201, JWH-081, and JWH-019, after LLE with a mixture of hexane: ethyl acetate (9:1), compared to those obtained either with a mixture of hexane/ethyl acetate (1/1, v/v) [45] or with n-hexane/ethyl acetate 90:10 (v/v) [42] and ethanol extraction [41], during LC–MS/MS.

**Phenylacetylindoles, Naphthoylpyrroles, Other Synthetic Cannabinoids:** A total of 10 studies have been conducted for the determination of 5 phenylacetylindoles in hair [[21], [22], [23],^25^,^29^,^41^,^42^,^45^,^46^,^48^]. The main extraction method applied is acidified methanol (0.1 M), with acceptable efficiency for most of the analytes, since the respective LODs were lower or comparable to those achieved after extraction with other LLE protocols [25,41,42,46,48].

Single-step methanol extraction [25] was the main hair extraction method used for JWH-030, JWH-147 and JWH-307, although it provided higher LODs compared to that obtained after LLE with other solvent combinations [29,42] or acidified methanol [21].

A total of 13 studies were performed in hair for the determination of 52 SCs (not included in the previous classes) using LC–MS/MS [21,25,29,[41], [42], [43],^45^,[48], [49], [50], [51]], GC–MS [46], and LC-HRMS [23]. The relative data are presented in Table 3.

The foremost extraction method applied is MeOH/ 0.1 M HCL, to define 26 of them [21], resulting in LODs comparable to those achieved with different solvents and extraction protocols [23,25,29,[41], [42], [43],^45^,[48], [49], [50], [51]]. Once again, LLE with hexane/ ethyl acetate before GC–MS analysis has obtained the highest LODs for the respective SCs [46].

### 2.4 Determination of Synthetic Opioids in hair

Synthetic opioids (SOs) act on the same brain targets as naturally occurring drugs of the opium poppy plant (e.g., morphine, heroin, and codeine) to produce analgesic (pain relief) effects [68]. The design of some SOs (e.g., methadone and fentanyl) progressed from therapeutic use to the clandestine synthesis of new fentanyl derivatives for the illicit market. Various fentanyl analogs (e.g., acetyl, furanyl-fentanyl, and carfentanyl) have shown particularly hazardous pharmacological effects [69,70].

A total of 12 studies have been conducted for the determination of 58 synthetic opioids, using LC–MS/MS [21,29,[52], [53], [54], [55], [56], [57],^59^,^60^], and LC-HRMS assays [30,58]. The relative data are presented in Table 4.

**Table 4:** Selected parameters of hair analysis for Synthetic Opioids.

**NPSs**	**Extraction Method**	**Method of Analysis**	**LOD (pg/mg)**	**LOQ (pg/mg)**	**LOI/** **LOR (pg/mg)**	**Concentrations- Clinical/forensic samples (pg/mg)**	**References**
**SYNTHETIC OPIOIDS**							
AH-7921	MeOH/ HCL 0.1 M at 40 °C, pulverization	LC-MS/MS	1		-/100		[21]
Acetyl fentanyl	M3® at 100 °C, SPE/Prime HLB cartridges	LC-MS/MS	0.004	0.012			[52]
	MeOH at 55^o^C	LC-MS-MS	0.2	0.6			[53]
	Acid digestion at 45^o^C, SPE/BondElute CertifyI	LC-MS/MS	0.2	0.5		1.0−1.4 (post-mortem cases)	[54]
	Acid digestion at 95 °C, LLE with hexane/EA	LC-MS/MS	0.3	1		1.0/0−2 cm	[55]
	MeOH at 55 °C	LC-MS/MS	Range 0.1−0.3			LOQ-3200	[56]
	MeOH/ ACN/ Acetate NH4 pulverization	LC-MS/MS	0.5	2			[57]
	MeOH at 55 °C	UHPLC-QTOF-HRMS	0.6	1.2		LOQ-230	[58]
	M3® reagent at 100 °C	LC-MS/MS	1	2			[29]
	MeOH/ HCL 0.1 M at 40 °C, pulverization	LC-MS/MS	1		-/100		[21]
Acetyl norfentanyl	M3® at 100 °C, SPE/Prime HLB cartridges	LC-MS/MS	0.003	0.011			[52]
	Acid digestion at 45^o^C, SPE/BondElute CertifyI	LC-MS/MS	0.2	0.5			[54]
	M3® reagent at 100 °C	LC-MS/MS	1	2			[29]
	MeOH/ ACN/ Acetate NH4 pulverization	LC-MS/MS	2.5	2			[57]
Acrylfentanyl	Acid digestion at 45^o^C, SPE/BondElute CertifyI	LC-MS/MS	0.2	0.5			[54]
	MeOH/ ACN/ Acetate NH4 pulverization	LC-MS/MS	0.5	2			[57]
	MeOH at 55 °C	UHPLC-QTOF-HRMS	0.6	1.2		LOQ	[58]
Alfentanil	M3® at 100 °C, SPE/Prime HLB cartridges	LC-MS/MS	0.005	0.017			[52]
	MeOH at 55^o^C	LC-MS-MS	0.1	0.3			[53]
	Acid digestion at 45^o^C, SPE/BondElute CertifyI	LC-MS/MS	0.2	0.5			[54]
	MeOH/ ACN/ Acetate NH4 pulverization	LC-MS/MS	0.5	2			[57]
	M3® reagent at 100 °C	LC-MS/MS	1	2			[29]
*α*-Methylfentanyl	MeOH at 55 °C	UHPLC-QTOF-HRMS	0.5	1.0			[58]
	MeOH/ ACN/ Acetate NH4 pulverization	LC-MS/MS	2.5	5			[57]
Benzoylfentanyl	MeOH at 55 °C	LC-MS/MS				621/4460/5870: proximal to distal hair sections (3 cm length each)	[59]
Butyryl fentanyl	M3® at 100 °C, SPE/Prime HLB cartridges	LC-MS/MS	0.005	0.015			[52]
	Acid digestion at 45^o^C, SPE/BondElute CertifyI	LC-MS/MS	0.2	0.5		2.0 (drug users hair samples)	[54]
	MeOH/ ACN/ Acetate NH4 pulverization	LC-MS/MS	0.5	2			[57]
	MeOH at 55 °C	UHPLC-QTOF-HRMS	0.6	1.2		54	[58]
	M3® reagent at 100 °C	LC-MS/MS	1	2		380	[29]
	MeOH/ HCL 0.1 M at 40 °C, pulverization	LC-MS/MS	1		-/100		[21]
Butyrylfentanyl Carboxy Metabolite	M3® at 100 °C, SPE/Prime HLB cartridges	LC-MS/MS	0.006	0.02			[52]
	Acid digestion at 45^o^C, SPE/BondElute CertifyI	LC-MS/MS	0.2	0.5			[54]
	M3® reagent at 100 °C	LC-MS/MS	1	2			[29]
Butyryl Norfentanyl	M3® at 100 °C, SPE/Prime HLB cartridges	LC-MS/MS	0.006	0.018			[52]
	Acid digestion at 45^o^C, SPE/BondElute CertifyI	LC-MS/MS	0.2	0.5			[54]
	M3® reagent at 100 °C	LC-MS/MS	1	2		160	[29]
b-Hydroxy-3-Methylfentanyl	MeOH/ ACN/ Acetate NH4 pulverization	LC-MS/MS	1	5			[57]
b-Hydroxyfentanyl	M3® at 100 °C, SPE/Prime HLB cartridges	LC-MS/MS	0.005	0.014			[52]
	Acid digestion at 45^o^C, SPE/BondElute CertifyI	LC-MS/MS	0.2	0.5			[54]
	M3® reagent at 100 °C	LC-MS/MS	1	2			[29]
	MeOH/ ACN/ Acetate NH4 pulverization	LC-MS/MS	1	5			[57]
b-Hydroxythiofentanyl	M3® at 100 °C, SPE/Prime HLB cartridges	LC-MS/MS	0.005	0.014			[52]
	Acid digestion at 45^o^C, SPE/BondElute CertifyI	LC-MS/MS	0.2	0.5			[54]
	MeOH/ ACN/ Acetate NH4 pulverization	LC-MS/MS	1	5			[57]
Carfentanil	MeOH at 55^o^C	LC-MS-MS	0.2	0.6			[53]
	M3® at 100 °C, SPE/Prime HLB cartridges	LC-MS/MS	0.006	0.019			[52]
	M3® reagent at 100 °C	LC-MS/MS	1	2			[29]
	MeOH/ ACN/ Acetate NH4 pulverization	LC-MS/MS	0.5	2			[57]
	MeOH at 55 °C	LC-MS/MS	Range 0.1−0.3			LOQ-1.5	[56]
	Acid digestion at 45^o^C, SPE/BondElute CertifyI	LC-MS/MS	0.2	0.5		1.2 (drug users hair samples)	[54]
	Acid digestion at 95 °C, LLE with hexane/EA	LC–MS/MS	0.8	2.5		9−12 Months after the Overdose/ S. B: 2−4 cm = 3.0 and S. A: 0−2 cm = 2.5	[55]
	MeOH at 55 °C	UHPLC-QTOF-HRMS	0.8	1.6			[58]
	MeOH at 55 °C	LC-MS/MS				54/114/166: (from proximal to distal hair section - 3 cm length each)	[59]
Cis-3-Methylfentanyl	MeOH/ ACN/ Acetate NH4 pulverization	LC-MS/MS	1	5			[57]
	MeOH/ HCL 0.1 M at 40 °C, pulverization	LC-MS/MS	1		-/100		[21]
Cis-3-Methyl Norfentanyl	M3® reagent at 100 °C	LC-MS/MS	1	2			[29]
Cyclopropyl Fentanyl	M3® at 100 °C, SPE/Prime HLB cartridges	LC-MS/MS	0.006	0.019			[52]
	MeOH at 55 °C	UHPLC-QTOF-HRMS	0.7	1.4		4.7	[58]
	M3® reagent at 100 °C	LC-MS/MS	1	2			[29]
	MeOH/ ACN/ Acetate NH4 pulverization	LC-MS/MS	1	5			[57]
Cyclopropyl Norfentanyl	M3® at 100 °C, SPE/Prime HLB cartridges	LC-MS/MS	0.006	0.018			[52]
	M3® reagent at 100 °C	LC-MS/MS	1	2			[29]
Despropionyl para-fluorofentanyl	M3® at 100 °C, SPE/Prime HLB cartridges	LC-MS/MS	0.005	0.015			[52]
	Acid digestion at 45^o^C, SPE/BondElute CertifyI	LC-MS/MS	0.2	0.5			[54]
	M3® reagent at 100 °C	LC-MS/MS	1	2			[29]
Fentanyl	M3® at 100 °C, SPE/Prime HLB cartridges	LC-MS/MS	0.003	0.013		2540−2,800	[52]
	MeOH at 55^o^C	LC-MS-MS	0.1	0.3		3−6	[53]
	MeOH at 55 °C	LC-MS/MS	Range 0.1−0.3			LOQ-8600	[56]
	Acid digestion at 45^o^C, SPE/BondElute CertifyI	LC-MS/MS	0.2	0.5		8.3−12.8 (post-mortem cases)	[54]
	Acid digestion at 95 °C, LLE with hexane/EA	LC-MS/MS	0.3	1		9−12 Months after the Overdose/ S. B:2−4 cm = 760 and S. A: 0−2 cm = 620	[55]
	MeOH/ ACN/ Acetate NH4 pulverization	LC-MS/MS	0.5	2		8.02	[57]
	MeOH at 55 °C	UHPLC-QTOF-HRMS	0.6	1.2		LOQ–1400	[58]
	M3® reagent at 100 °C	LC-MS/MS	1	2		2800−3200	[29]
Fentanyl-D5	Acid digestion at 45^o^C, SPE/BondElute CertifyI	LC-MS/MS	0.2	0.5			[54]
Furanyl Fentanyl	M3® at 100 °C, SPE/Prime HLB cartridges	LC-MS/MS	0.005	0.016			[52]
	MeOH at 55^o^C	LC-MS-MS	0.1	0.3		44	[53]
	MeOH at 55 °C	LC-MS/MS	Range 0.1−0.3			LOQ-590	[56]
	Acid digestion at 45^o^C, SPE/BondElute CertifyI	LC-MS/MS	0.2	0.5		136.7−195.8 (post-mortem cases)	[54]
	MeOH at 55 °C	UHPLC-QTOF-HRMS	0.6	1.2		LOQ-6300	[58]
	Acid digestion at 95 °C, LLE with hexane/EA	LC-MS/MS	0.8	2.5		9−12 Months after the Overdose/ S. B: 2−4 cm = 500 and S. A: 0−2 cm = 310	[55]
	M3® reagent at 100 °C	LC-MS/MS	1	2			[29]
	MeOH/ ACN/ Acetate NH4 pulverization	LC-MS/MS	1	5			[57]
Furanylethyl Fentanyl	M3® at 100 °C, SPE/Prime HLB cartridges	LC-MS/MS	0.003	0.014			[52]
	M3® reagent at 100 °C	LC-MS/MS	1	2			[29]
Furanyl Norfentanyl	Basic digestion at 95 °C, LLE with hexane/EA	LC-MS/MS	0.003	0.012			[42]
	Acid digestion at 45^o^C, SPE/BondElute CertifyI	LC-MS/MS	0.2	0.5			[54]
	M3® reagent at 100 °C	LC-MS/MS	1	2			[29]
Hydrocodone	MeOH at 55^o^C	LC-MS-MS	0.1	0.3		13−71	[53]
	MeOH at 55 °C	LC-MS/MS	Range 0.1−0.3			LOQ-12,600	[56]
Isobutyryl Fentanyl	MeOH/ ACN/ Acetate NH4 pulverization	LC-MS/MS	2.5	5			[57]
Methoxyacetyl fentanyl	M3® at 100 °C, SPE/Prime HLB cartridges	LC-MS/MS	0.005	0.016			[52]
	M3® reagent at 100 °C	LC-MS/MS	1	2			[29]
	MeOH/ ACN/ Acetate NH4 pulverization	LC-MS/MS	0.5	2			[57]
	Acid digestion at 45^o^C, SPE/BondElute CertifyI	LC-MS/MS	0.5	1		259.9−479.6 (post-mortem cases)	[54]
	Acid digestion at 95 °C, LLE with hexane/EA	LC-MS/MS	0.3	1		9−12 Months after the Overdose/ S. B: 2−4 cm = 600 and S. A: 0–2 cm = 500	[55]
Methoxyacetyl Norfentanyl	Acid digestion at 45^o^C, SPE/BondElute CertifyI	LC-MS/MS	0.5	1		17.1- 32.7 (post-mortem cases)	[54]
	MeOH at 55^o^C	LC-MS/MS	0.005	0.016			[53]
	M3® reagent at 100 °C	LC-MS/MS	1	2			[29]
*N*-Desmethyl U-47,700	MeOH/ ACN/ Acetate NH4 pulverization	LC-MS/MS	0.5	2			[57]
Norcarfentanil	MeOH/ ACN/ Acetate NH4 pulverization	LC-MS/MS	0.5	2			[57]
Norfentanyl	MeOH at 55^o^C	LC-MS-MS	0.1	0.3			[53]
	M3® at 100 °C, SPE/Prime HLB cartridges	LC-MS/MS	0.005	0.015		15.1−149	[52]
	MeOH/ ACN/ Acetate NH4 pulverization	LC-MS/MS	1	5			[57]
	MeOH at 55 °C	LC-MS/MS	Range 0.1−0.3			LOQ-320	[56]
	Acid digestion at 45^o^C, SPE/BondElute CertifyI	LC-MS/MS	0.2	0.5			[54]
	MeOH at 55 °C	UHPLC-QTOF-HRMS	1.2	2.4		3.5−600	[58]
Ocfentanil	Acid digestion at 45^o^C, SPE/BondElute CertifyI	LC-MS/MS	0.2	0.5		0.9 (drug users hair samples) 4.1−11.1 (post-mortem cases)	[54]
	MeOH at 55 °C	UHPLC-QTOF-HRMS	0.4	0.8			[58]
	MeOH/ ACN/ Acetate NH4 pulverization	LC-MS/MS	0.5	2			[57]
Oxycodone	MeOH at 55^o^C	LC-MS-MS	1.5	4.5		13−780	[53]
	MeOH at 55 °C	LC-MS/MS	1.5			LOQ-25,700	[56]
*para/ortho*-Fluorofentanyl	MeOH/ ACN/ Acetate NH4 pulverization	LC-MS/MS	2.5	5			[57]
PFBF	Acid digestion at 45^o^C, SPE/BondElute CertifyI	LC-MS/MS	0.2	0.5			[54]
	MeOH/ ACN/ Acetate NH4 pulverization	LC-MS/MS	2.5	5			[57]
Phenylacetyl fentanyl	M3® at 100 °C, SPE/Prime HLB cartridges	LC-MS/MS	0.005	0.015			[52]
	Acid digestion at 45^o^C, SPE/BondElute CertifyI	LC-MS/MS	0.5	1			[54]
	M3® reagent at 100 °C	LC-MS/MS	1	2			[29]
Remifentanil acid	MeOH/ ACN/ Acetate NH4 pulverization	LC-MS/MS	2.5	5			[57]
Remifentanil	MeOH at 55^o^C	LC-MS-MS	0.3	0.9			[53]
	Acid digestion at 45^o^C, SPE/BondElute CertifyI	LC-MS/MS	0.2	0.5			[54]
	MeOH/ ACN/ Acetate NH4 pulverization	LC-MS/MS	0.5	2			[57]
Sufentanil	M3® at 100 °C, SPE/Prime HLB cartridges	LC-MS/MS	0.006	0.019			[52]
	MeOH at 55^o^C	LC-MS-MS	0.3	0.9			[53]
	MeOH/ ACN/ Acetate NH4 pulverization	LC-MS/MS	0.5	2		S1: 0−3 cm: 183.91, S2: 3−6 cm: 131.68,S3: 6 −9 cm: 31.48	[57]
	Acid digestion at 45^o^C, SPE/BondElute CertifyI	LC-MS/MS	0.5	1			[54]
THFF	Acid digestion at 45^o^C, SPE/BondElute CertifyI	LC-MS/MS	0.2	0.5		1.3 (drug users hair samples)	[54]
	MeOH/ ACN/ Acetate NH4 pulverization	LC-MS/MS	0.5	2			[57]
Thiofentanyl	MeOH/ ACN/ Acetate NH4 pulverization	LC-MS/MS	2.5	5			[57]
Tramadol	MeOH at 55^o^C	LC-MS-MS	0.1	0.3		2.0−3,700	[53]
	MeOH at 55 °C	LC-MS/MS	Range 0.1−0.3			LOQ-34,700	[56]
	M3® reagent at 100 °C	LC-MS/MS	5	10		12,300−15,000	[29]
Trans-3-Methylfentanyl	MeOH/ HCL 0.1 M at 40 °C, pulverization	LC-MS/MS	1		-/100		[21]
	MeOH/ ACN/ Acetate NH4 pulverization	LC-MS/MS	2.5	5			[57]
Trans-3-Methylnorfentanyl	Acid digestion at 45^o^C, SPE/BondElute CertifyI	LC-MS/MS	0.2	0.5		35.9 (In subject S29 the metabolite of 3-methyl fentanyl was identified. Unfortunately, due to the lack of reference standard, the presence of parent drug was not confirmed/ Positive results of hair samples collected from drug users hair samples))	[54]
	M3® reagent at 100 °C	LC-MS/MS	1	2			[29]
U-47,700	MeOH at 55^o^C	LC-MS-MS	0.1	0.3			[53]
	MeOH at 55 °C	LC-MS/MS	Range 0.1−0.3			LOQ-420	[56]
	MeOH/ ACN/ Acetate NH4 pulverization	LC-MS/MS	0.5	2			[57]
	MeOH/ HCL 0.1 M at 40 °C, pulverization	LC-MS/MS	10		-/100		[21]
	MeOH at 55 °C pulverization	LC-MS/MS				5700	[60]
U-48,800	MeOH/ ACN/ Acetate NH4 pulverization	LC-MS/MS	0.5	2			[57]
U-51,754	MeOH/ ACN/ Acetate NH4 pulverization	LC-MS/MS	0.5	2			[57]
U-50,488	MeOH/ ACN/ Acetate NH4 pulverization	LC-MS/MS	1	5			[57]
Valeryl fentanyl	MeOH/ ACN/ Acetate NH4 pulverization	LC-MS/MS	2.5	5			[57]
Valeryl fentanyl carboxy metabolite	M3® at 100 °C, SPE/Prime HLB cartridges	LC-MS/MS	0.007	0.021			[52]
	Acid digestion at 45^o^C, SPE/BondElute CertifyI	LC-MS/MS	0.5	1			[54]
	M3® reagent at 100 °C	LC-MS/MS	1	2			[29]
W-18	MeOH/ HCL 0.1 M at 40 °C, pulverization	LC-MS/MS	1		-/100		[21]
	MeOH/ ACN/ Acetate NH4 pulverization	LC-MS/MS	2.5	5			[57]
3- fluorofentanyl	Incubation at 95 °C, LLE with Hept/EA, DCM/Isopropanol	LC-HRMS- Orbitrap	1				[30]
3(meta)-fluorofentanyl	Acid digestion at 95 °C, LLE with hexane/EA	LC-MS/MS	0.8	2.5		9–12 Months after the Overdose/ Segment B: 2−4 cm = 80 and Segment A: 0–2 cm = 25	[55]
3-Methylthiofentanyl	MeOH/ ACN/ Acetate NH4 pulverization	LC-MS/MS	1	5			[57]
4-ANPP	M3® at 100 °C, SPE/Prime HLB cartridges	LC-MS/MS	0.006	0.018		10.4−11.2	[52]
	MeOH at 55^o^C	LC-MS-MS	0.1	0.3		1−2	[53]
	MeOH at 55 °C	LC-MS/MS	Range 0.1−0.3			LOQ-1400	[56]
	Acid digestion at 45^o^C, SPE/BondElute CertifyI	LC-MS/MS	0.2	0.5			[54]
	MeOH/ ACN/ Acetate NH4 pulverization	LC-MS/MS	0.5	2			[57]
	MeOH at 55 °C	UHPLC-QTOF-HRMS	0.7	1.4		1.4−230	[58]
	M3® reagent at 100 °C	LC-MS/MS	1	2		7	[29]
4-Fluorobutyrfentanyl	MeOH at 55 °C	UHPLC-QTOF-HRMS	0.2	0.4		5.2−180	[58]
	MeOH at 55 °C	LC-MS/MS				4/152/719: proximal to distal hair sections (3 cm length each)	[59]
4-Fluoroisobutyryl fentanyl	MeOH/ ACN/ Acetate NH4 pulverization	LC-MS/MS	2.5	5			[57]

Extraction with MeOH/ACN/ammonium acetate was mainly utilized for the determination of 37 SOs, achieving comparable and considerably low LODs for 11 of them [57].

Generally, similar LODs were achieved for the determination of several SOs, after extraction with different organic solvents [53,56,58,60], or combinations of organic solvents [21,29,30,55,57], or SPE [54].

Notable, one SPE protocol [52] resulted in considerably lower LODs for the tested analytes than those achieved with LLEs protocols indicating that improvements in SPE matrices could result in more efficient extractions and lower LODs.

### 2.5 Determination of synthetic tryptamines and other NPS in hair

Over the last few years, synthetic tryptamine analogs (STs) show a growing demand among drug users. STs can offer increased potencies compared to natural tryptamines as a result of a functional group modification, specifically from decarboxylation of the amino acid tryptophan [71].

A total of five studies have been conducted for the determination of ten tryptamines in hair using LC–MS/MS [21,29,61], and LC-HRMS assays [30,62]. The relative data are presented in Table 5. Three of these studies [21,29,30,62], performed simultaneously analysis of tryptamines with other NPS classes.

**Table 5:** Selected parameters of hair analysis for Synthetic Tryptamines and other NPS.

**NPSs**	**Extraction Method**	**Method of Analysis**	**LOD (pg/mg)**	**LOQ (pg/mg)**	**LOI/LOR (pg/mg)**	**Concentrations- Clinical/forensic samples (pg/mg)**	**References**
**TRYPTAMINES**							
AcO DMT	M3® reagent at 100 °C	LC-MS/MS	2	6			[29]
DMT	MeOH/ HCL 0.1 M at 40 °C, pulverization	LC–MS/MS	10		-/100		[21]
4-AcO-DIPT	M3® reagent at 100 °C	LC-MS/MS	2	6			[29]
4-OH DET	M3® reagent at 100 °C	LC-MS/MS	2	6			[29]
5-MeO-AMT	M3® reagent at 100 °C	LC-MS/MS	2	6		70	[29]
5-MeO-DALT	Incubation at 95 °C, LLE with Hept/EA, DCM/Isopropanol	LC-HRMS- Orbitrap	1		50/-		[30]
	M3® reagent at 100 °C	LC-MS/MS	2	6			[29]
5-MeO-DiPT	aq.HCOOH pulverization at 4 °C	LC-MS/MS	0.05	LLOQ: 0.1		0.2−7532.5	[61]
5-MeO-DPT	M3® reagent at 100 °C	LC-MS/MS	2	6			[29]
5-MeO-DMT	MeOH at 60 °C	LC-MS/MS, LC-HRMS	25	LLOQ: 100		1990−3390	[62]
5-MeO-MIPT	M3® reagent at 100 °C	LC-MS/MS	2	6			[29]
**Other NPS**							
Benzoylecgonine	PLE, SPE/C18 cartridge	LC-MS-MS	1	3.5			[28]
	Incubation at 95 °C, LLE with Hept/EA, DCM/Isopropanol	LC-HRMS- Orbitrap	5		50/-		[30]
	MeOH/ HCL 0.1 M at 40 °C, pulverization	LC-MS/MS	50		-/100		[21]
Deschloroketamine	Aq.HCOOH 0.1 M at 40 °C	LC-MS/MS	10	50			[63]
	Aq.HCOOH 0.1 M at 40 °C	LC-HRMS	50				[63]
Diphenidine	MeOH/ HCL 0.1 M at 40 °C, pulverization	LC–MS/MS	1		-/100		[21]
	MeOH at 55 °C	LC-MS/MS	3.4				[20]
EPH	MeOH/ HCL 0.1 M at 40 °C, pulverization	LC-MS/MS	10		-/100		[21]
Ketamine	PLE, SPE/C18 cartridge	LC-MS-MS	2.5	8			[28]
	Incubation at 95 °C, LLE with Hept/EA, DCM/Isopropanol	LC-HRMS- Orbitrap	5		50/-		[30]
	M3® reagent at 100 °C	LC-MS/MS	5	12		80- 27,300	[29]
	MeOH/ HCL 0.1 M at 40 °C, pulverization	LC-MS/MS	10		-/100		[21]
	MeOH/ HCL 1%	GC/MS	500	500			[32]
Mescaline	PLE, SPE/C18 cartridge	LC-MS-MS	3.7	13			[28]
	MeOH/ HCL 0.25 M at 50 °C	GC/MS	9	40			[19]
	MeOH/ HCL 0.1 M at 40 °C, pulverization	LC-MS/MS	50		-/100		[21]
Methylphenidate	MeOH/ HCL 0.1 M at 40 °C, pulverization	LC-MS/MS	10		-/100		[21]
Methoxpropamine	Aq.HCOOH 0.1 M at 40 °C	LC-MS/MS	10	50			[63]
	Aq.HCOOH 0.1 M at 40 °C	LC-HRMS	50				[63]
MPA	MeOH/ HCL 0.1 M at 40 °C, pulverization	LC-MS/MS	10		-/100		[21]
	Incubation at 95 °C, LLE with Hept/EA, DCM/Isopropanol	LC-HRMS- Orbitrap	50		50/-		[30]
Norketamine	M3® reagent at 100oC	LC-MS/MS	5	12		40−8400	[29]
	MeOH/ HCL 0.1 M at 40 °C, pulverization	LC-MS/MS	10		-/100		[21]
	MeOH/ HCL 0.25 M at 50 °C	GC/MS	21	80		0.34	[19]
	MeOH/ HCL 1%	GC/MS	250	500			[32]
PCP	MeOH/ HCL 0.1 M at 40 °C, pulverization	LC-MS/MS	1		-/100		[21]
	PLE, SPE/C18 cartridge	LC-MS-MS	1.5	2.4			[28]
2-fluoro-deschlotoketamine	Aq.HCOOH 0.1 M at 40oC	LC-MS/MS	10	50			[63]
	Aq.HCOOH 0.1 M at 40 °C	LC-HRMS	50				[63]
	MeOH at 60 °C	LC-MS/MS, LC-HRMS	25	LLOQ: 100			[62]
3-MeO-PCP	MeOH/ HCL 0.1 M at 40 °C, pulverization	LC-MS/MS	50		-/100		[21]
3-methoxyeticyclidine (3-MeO-PCE)	MeOH at 60 °C	LC-MS/MS, LC-HRMS	25	LLOQ: 100		1610- 3610	[62]
4-MeO-PCP	MeOH at 55 °C	LCMS/MS	9	1.8			[20]
	MeOH/ HCL 0.1 M at 40 °C, pulverization	LC-MS/MS	50		-/100		[21]

The LODs were comparable for the different tryptamines determined. Interestingly, methanol was not the main solvent for the extraction of tryptamines from hair. The different extraction protocols applied resulted in comparable LODS for the different compounds analysed.

A total of nine studies have been conducted for the determination of 15 other NPS in hair, using LC–MS/MS [20,21,28,63], GC–MS [19,32], and LC-HRMS assays [30,63]. The relative data are presented in Table 5. Overall, the leading extraction method applied was again acidified methanol (0.1 M) [21].

## 3 Aspects of NPS hair extraction

The different applied hair extraction procedures have focused on isolating from the hair matrix, certain NPS, of the same or different classes, with the highest possible efficiency. It is known that the keratinized hair shaft has a complex, multi-compartment structure and the drug incorporation in hair is a function of the acidity/basicity of the compound, its lipophilicity, and its affinity to melanin [72]. On the other hand, the main factors that influence drug incorporation in hair, affect the drug extraction from hair, as well [73]. Therefore, the extraction procedure poses several issues most relevant to the complexity of this matrix.

Of main concern to the toxicologists is the removal of the external contaminations from hair, consisting of organic and inorganic chemicals that have been deposited to the hair shafts, by applying appropriate washing steps. The hair washing steps are usually applied according to the suggested guidelines which states further that external contamination must be considered to interpret findings, while researchers should evaluate the efficiency of washing procedures [73,74]. Nevertheless, some of the reviewed studies herein, reported extensive washing procedures, and others none, indicating the different viewpoints for the necessity of this step, especially in respect to differentiate the active drug use from external drug deposition onto hair.

Several extraction methods have been applied to isolate NPS from hair (such as methanolic extraction, LLE, or SPE, in ultrasonic and/or heating blocks, under different conditions), depending on the chemical properties of the analytes. The dominant extraction method applied for the determination of most NPS classes was acidified methanol (with various concentrations of hydrochloride). This preference should be attributed, firstly, to its ability to extract from hair very diverse classes of NPS, and secondly, to the simplicity of the relevant procedures, as compared to SPE, or other LLEs. However, the extraction with acidified methanol presents the disadvantages of yielding lower drug recoveries compared to other procedures, and of resulting in a high degree of contamination from hair matrix (matrix effect) [72,73]. In fact, matrix decontamination is one of the main limitations in hair testing, with an impact on extraction efficiencies and on the LODs/LOQs of the analytes. Although not within the scope of this review, we comment that the extraction efficiencies of the various NPS analysis methodologies, as expressed by precision and accuracy, were considered satisfactory [75].

Our review data depicted on Table 1, Table 2, Table 3, Table 4, Table 5 have revealed that NPS of the same chemical class had similar LODs and range of identification when extracted from hair with the same medium and detected with the same method, as expected. Additionally, our data made apparent that the LODs, LOQs and ranges of various NPS groups determined in hair, with different analysis protocols, were comparable (being all in the low pg/mg level). These data indicate that modern NPS hair analysis ensures high selectivity and sensitivity of detection of different analytes. It is worth mentioning that different NPS with very diverse chemical structures were efficiently extracted from hair, with acidified methanol; proving acidified methanol as a generic extractor.

Last but not least finding of our review was that the NPS hair levels measured in real forensic/ clinical cases were significantly higher than the respective LODs. The NPS hair analysis of real cases reported the detection of 80 NPS (out of the 280 targeted NPS) with the respective developed methods for NPS hair analysis (only five of the reviewed publications don’t report application in real cases [21,23,28,31,37]). These findings indicate that the existing methods enable adequate identification and measurement of these compounds in hair from possible NPS (ab)users. However, it should be underlined that one of the reviewed studies had set LOR as the cut-off analyte concentration that can discriminate passive exposure from active incorporation of NPS in hair (it was set at the level 100 pg/mg of hair and being at least 10fold higher than the respective LODs); subsequently cases with NPS concentrations higher than the predefined LOR were considered positive [21]. Although this LOR value was set arbitrarily, the relevant consideration is in accordance with the recently expressed concerns on the possibility of misinterpretations of the very low drug concentrations in hair [76]. Specifically, for NPS more concerns could arise from the absence of specific guidelines for their analysis, the absence of official cut-offs to discriminate consumption from contamination, and from their unknown pharmacology which probably would make necessary the establishment of lower cut-offs in certain cases. Probably, future research could advance the progress of more efficient extraction mixtures or micro extraction methods of diverse NPS from hair [[77], [78], [79]]. Undoubtedly, an improved universal extraction protocol of NPS from hair will advance the aim of studying the epidemiology of NPS among drug abusers.

## 4 Aspects of the NPS detection methods

The most crucial issue on NPS hair analysis is the accurate compound detection and identification. The objective measures of identity are assured by the various available mass spectrometry techniques which enable the definitive identification of analytes, reducing to a minimum, or ideally, eliminating, the number of false-positive and false-negative identifications. NPS hair analysis was exclusively performed by MS techniques, after a chromatographic (LC or GC) separation.

All but one reviewed methods herein, concerned targeted analysis of NPS in hair (mainly on low resolution mass spectrometers, LRMS) allowing the detection of few to several NPS of one class or of different classes (up to 132 NPS which is the largest number of NPS so far [21]. The “targeted” analysis strategy achieves the definitive identification and confirmation of an unknown analyte, by “fitting” selected MS data (*m/z* values of molecular ions, relative abundances of fragment ions, etc.) and relevant chromatographic parameters (such as the retention time of the analyte) with, either the MS data of a reference standard analysed under the same conditions as the unknown, in accordance with the suggested up to date relevant guidelines, or the data form MS/MS spectral libraries. Nevertheless, a serious limitation of the targeted NPS identification with LRMS is the lack of verified reference standards for newly identified NPS and metabolites.

One the other hand, the HRMS screening approach can overcome this limitation and therefore it became gradually more popular than LRMS for comprehensive drug screening, including NPS analysis. Worth mentioning advantages of HRMS, including high resolution and high mass accuracy, both of which increase the confidence of compound identification, and selectivity of the method, allowing the confirmation of the NPS molecular formula; even in identifying the minor mass differences that are often present in NPS molecules [[80], [81], [82]]. Furthermore, HRMS could prove beneficial in identifying structural clusters of potential novel toxic metabolites of NPS [[83], [84], [85]].

However, comprehensive HRMS-based screening and confirmatory methods for NPS hair analysis are reported to a limited number of studies, mainly because they need standardized spectral libraries for screening and identification of compounds present in a sample [23,30,38]. Some attempts [23,86] succeeded to construct MS/MS spectral libraries including large numbers of NPS and metabolites. Initially, Montesano and colleagues described the development of a broad screening technique for NPS that included the use of an in-house MS/MS spectral library for 300 NPS and known metabolites [23]. More recently, the development of a comprehensive compound database for 875 unique chemical entities considered as possible NPS, in addition to a full HRMS MS/MS spectral library for 252 of these compounds was reported [86].

Although these recent trends in NPS analysis, presently, HRMS instrumentation does not seem to be a replacement for standard LRMS which are a commonplace worldwide for routine basic toxicology applications. Routine toxicology procedures need to fulfill standard guidelines which are not set yet in the forensic field for not targeted analyses provided by the HRMS instruments. However, we are of opinion that hair analysis by HRMS could be advantageous for conducting any NPS and metabolite identification, in the aim to study the prevalence and spread of NPS use in the community.

## 5 Conclusions

Initially, NPS hair analysis was performed by using the established analytical methodologies for drugs of abuse hair analysis which were validated as suggested by official organizations. However, the quickly changing chemical structures of new NPS delivered in the illegal drug markets, the unknown pharmacology of new NPS, and the possibility some of them to have high potency, which could lead to emergency hospitalizations and/or deaths has challenged their analysis in biological specimens. Therefore, the improvement of analytical strategies, and the development of alternative and innovative analytical methodologies became a necessity. The relative research has focused, mainly, on improving the NPS extraction from hair and, the detection techniques of extracted analytes.

The analytical protocols reviewed herein for NPS hair analysis showed continuously growing trends to identify as many NPS as possible; the extraction methods seem to have a limited potential to improve, while the various mass spectroscopic techniques and relevant instrumentation used for NPS detection and identification provide an enormous field for development and application. Future research in the field could progress NPS hair analysis and aim the monitoring of NPS expansion and extent of use worldwide.

## Authors’ statement

The authors declare that they have contributed to the manuscript as follows:

• DF has written the original manuscript according to VB’s suggestions;
• VB has designed the manuscript, supervised the writing and wrote the critical discussion of the subject.

## Declaration of Competing Interest

The authors declare that they have no known competing financial interests or personal relationships that could have appeared to influence the work reported in this paper.
